# Solvation Energetic Costs of Cognate Binding Site Formation

**DOI:** 10.1021/acs.jcim.5c01432

**Published:** 2025-08-15

**Authors:** Yeonji Ji, Vjay Molino, Steven Ramsey, Tom Kurtzman

**Affiliations:** † Ph.D. Program in Biochemistry, The Graduate Center, 14772 City University of New York, New York, New York 10016, United States; ‡ Department of Chemistry, Lehman College, 14772 City University of New York, Bronx, New York 10468, United States; § Ph.D. Programs in Biology & Chemistry, The Graduate Center, 14772 City University of New York, New York, New York 10016, United States

## Abstract

Structural fluctuations of proteins can reveal alternate binding site conformations or cryptic pockets that may be exploited to discover novel, tightly bound chemical compounds. While significant effort has been dedicated to the exploration of protein conformational space, the thermodynamic role of solvation and how it is coupled to a protein’s structural fluctuations, particularly in binding site formation, has not been well characterized. In this study, we examine how binding site solvation energetics differ between unligated *rigid* cavities restrained about their ligand-bound conformations and the same cavities with *flexible* side chains free to explore conformational space in molecular dynamics simulations. We find that, on average, the solvation energy of *flexible* binding sites is significantly more favorable, 14.4 kcal/mol, than that of their *rigid* counterparts. Our analysis of the solvation reveals that this energetic discrepancy is driven by the *flexible* binding sites structuring themselves to form more energetically favorable protein–water hydrogen bonds than in the *rigid* cavities. The substantial solvation energetic cost for a *flexible* protein to adopt conformations that are complementary to cognate ligands (We use the term *cognate ligand* to refer to the ligand in the cocrystallized complex in the corresponding pdb entry. The term *cognate structure* refers to the experimentally determined protein–ligand complex containing this ligand.) led us to hypothesize that there may be little overlap between binding site side chain configurations of unligated proteins and those of ligated proteins that have structured their cavities to optimize protein–ligand interactions. We therefore investigate the configurations of *flexible* binding site side chains in unligated systems and find that in some proteins, they do not sample conformations that are complementary to their cognate ligands in molecular dynamics simulations. Notably, we identify a class of binding sites characterized by highly enclosed cavities with bidentate ligand interactions that are especially prone to this solvation-induced conformational occlusion, in which there is little to no overlap in the conformational landscapes of ligated and unligated binding cavities. We discuss how understanding the interplay between solvation energetics and protein structural fluctuations can inform the development of methods aimed at discovering alternative binding pockets, improve methods such as WaterMap and GIST that estimate the contribution to binding affinity of displacing water upon ligand binding, and can be used to inform bindability assessments of revealed cryptic pockets.

## Graphical abstract

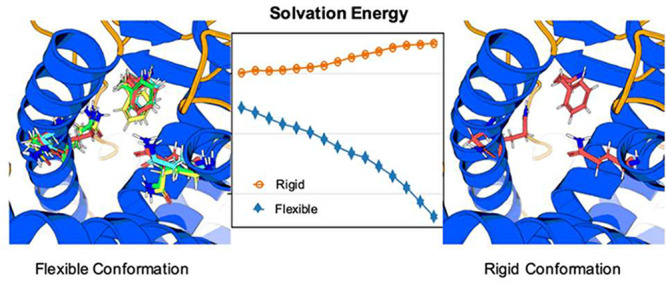

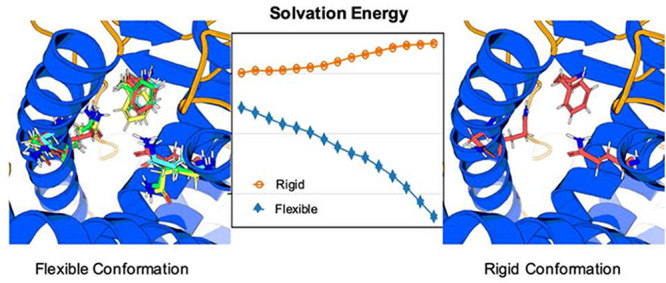

## 1 Introduction

The solvation contribution to the binding affinity between a small molecule and protein is fully described by the difference in solvation free energy between an initial state, in which the protein and ligand are unbound, and a final state, in which the ligand and protein are bound (Figure 1).^1^ In the initial state, the protein and ligand are independently solvated and are more flexible than in the final state, in which the protein and ligand are effectively limited to conformations that are complementary to each other (Figure 1). Solvation mapping methods based upon Inhomogeneous fluid Solvation Theory (IST),^2,3^ such as WaterMap,^4^ Grid Inhomogeneous Solvation Theory (GIST),^5,6^ and Solvation Structural and Thermodynamic Mapping (SSTMap),^7^ have been used to estimate the free energy of displacing solvent from the binding cavity upon ligand recognition. The formulation of IST relies upon Percus’ source particle method, which applies to a rigid conformation of the solute.^8^ As a result, computational methods aimed at estimating the thermodynamic contribution of solvent displacement from the binding cavity generally rely on solvation thermodynamic estimates of *rigid* systems, rather than flexible ones, therefore approximating the solvation contribution as the difference between the solvation properties of the models shown in Figure 1c,d.

**Figure 1:**
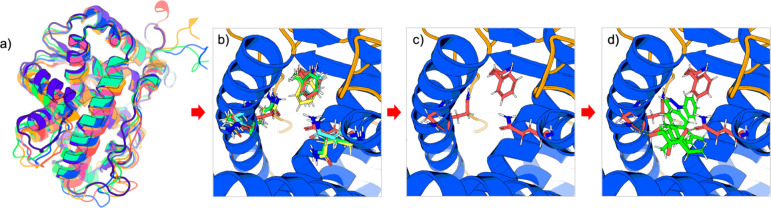
Thermodynamic path of binding between the glucocorticoid receptor (GCR, PDB id: 3BQD^26^) and the ligand DAY broken down into four states: (a) initial state in which the ligand and protein are fully flexible and unbound (ligand not shown); (b) intermediate state with the protein backbone restrained about the cognate ligand-bound conformation, while the side chains remain flexible; (c) second intermediate state in which both the backbone and side chains are restrained about their cognate ligand-bound conformation; and (d) final protein–ligand complex. Differently colored backbones and side chains are representative of their conformations in each state.

Based on our prior work,^9,10^ which describes the strong coupling between host system conformations and the thermodynamics of solvation, we hypothesized that structural fluctuations of protein binding sites are strongly coupled to solvation thermodynamics. Here, using GIST and SSTMap methods, we computationally investigate the structure and energetics of binding cavity water in *flexible* simulations, in which the side chains are unrestrained, and compare them to the solvation of *rigid* binding cavities, in which the side chains are restrained about their cognate ligand-bound structures. This comparison focuses on part of a thermodynamic end-state, in which one state (Figure 1b) is an unbound protein that has already adopted the cognate backbone structure but has freely moving side chains (*flexible*), and the other state (Figure 1c) is one in which the side chains are restrained about their cognate ligand-bound configuration (*rigid*). We find that solvation energy penalties to cognate structure formation for these end-states are significant for all 34 systems investigated, with average energetic costs computationally estimated to be 14.43 kcal/mol. We further investigate how the water structure varies between the *rigid* and *flexible* systems and describe how water complementarity to the protein surface (water’s ability to form complementary hydrogen bonds with the protein surface) and enhancement or frustration of water networks solvating the binding site contribute to the substantial solvation cost for the *flexible* system to adopt the *rigid* conformations consistent with the cognate ligand-bound conformation. Consistent with our prior studies, we identify cognate binding site topographies, namely, bidentate ligands in highly enclosed binding pockets, that frustrate the binding site water structure, which contributes to these large solvation penalties.^11^

The substantial solvation energetic cost associated with *flexible* systems adopting the cognate ligand-bound conformations observed in *rigid* systems prompted us to investigate whether this cost hinders side chains from exploring ligand-complementary conformations in the absence of a bound ligand. In molecular dynamics simulations, we observed that certain side chains that form hydrogen bonds with the ligand in the crystal structure do not adopt ligand-complementary conformations in unligated *flexible* simulations. This observation is consistent with the induced fit model. We discuss the implications of this finding in the context of computational methodologies aimed at exploring alternative conformations and identifying cryptic pockets capable of binding small-molecule ligands in the Section 4.

Finally, a number of methods have been developed to estimate or map out solvation thermodynamics on the surfaces of proteins, including 3D-RISM,^12–14^ GIST, WaterMap, Szmap,^15,16^ and others.^17–21^ Though these tools have provided valuable insight into target binding sites and enabled drug design ideas, the statistical mechanical formulation that connects the solvation distributions to solvation thermodynamics relies on the assumption that the target binding site is rigid. While these methods can be applied to flexible systems as some have done,^22^ the rigorous connection to solvation thermodynamics is then lost. For this reason, most applications of solvation mapping methods that have been used to estimate the contribution of solvent displacement upon ligand binding have relied on the solvation thermodynamics of the *rigid* cavities. The results presented here suggest that this is a poor approximation and, on average, vastly overestimates the solvation displacement contribution to binding affinity. This highlights the need for methods that provide better approaches to estimate binding site solvation thermodynamics, accounting for binding site flexibility.

The structure of the paper is as follows:

Section 2 outlines the protocols for protein preparation, molecular dynamics simulations, and mapping of the solvation structure and energetics. Section 3 presents an analysis of the solvation energetic penalties associated with forming *rigid* structures from *flexible* binding site conformations across 34 proteins. It also examines how the water structure and its complementarity to the protein surface differ between the *rigid* and *flexible* systems. Finally, for three selected systems (PDB ids: 2GTK,^23^ 3CCW,^24^ and 3BIZ^25^), we investigate the reorganization of side chains in the *flexible* systems compared to the *rigid* systems and detail how these are coupled to significant changes in solvation structure and energetics. Importantly, we find that in some *flexible* systems, side chains rarely, if ever, sample conformations that would be complementary to the known bound ligands.

We conclude the paper with a discussion of two important ramifications of the solvation energetic costs reported here. First, these costs, observed across a diverse set of systems, suggest that such conformations are inherently disfavored in the absence of ligands, limiting their sampling in both the simulation and the experiment when the protein is unligated. This has important ramifications for computational strategies aimed at identifying cryptic pockets or an alternate binding site conformation. Second, our findings challenge a common assumption in solvation mapping methodologies, i.e., the binding cavity is rigid, highlighting how this assumption can lead to an overestimation of the favorability of water displacement upon ligand binding.

## 2 Methods

We performed explicit solvent molecular dynamics (MD) simulations using Amber20 ^27^ and Amber22 ^28^ on a subset of 34 proteins from the Database of Useful Decoys–Enhanced (DUD-E).^29^ In one set of simulations, all heavy atoms were harmonically restrained about a protein structure that was minimized in the presence of the cognate ligand (termed *rigid*), and in a second set of simulations, the side chain heavy atoms were left unrestrained (termed *flexible*). In both sets of simulations, the proteins were simulated fully solvated with no ligand.

### 2.1 System Preparation and MD Simulation

#### 2.1.1 Selection and Preparation of Proteins

We investigated the subset of protein–ligand complexes from the DUD-E database in which the cognate ligand made three or more hydrogen bonds with binding site residues (34 of 102 systems). The structures of the 34 systems were obtained from the Protein Data Bank (PDB).^30^ All nonprotein atoms except those of the cognate ligand and water were removed. For proteins with multiple domains, we retained only the chains necessary to investigate the binding site. The PDB ID of each system, chain, and cognate ligand ID used for further steps can be found in Table S1 of the Supporting Information. The complexes were then prepared using the Protein Prep Wizard^31,32^ in Schrodinger Maestro^33^ with default settings. This step assigned protonation states, filled gaps in the structure, optimized side chain orientations, and capped the protein termini with *N*-acetyl and *N*-methyl amide groups. Water molecules farther than 5 Å from the ligand were removed. Protein atoms were parametrized with tleap^28^ using the ff14SB force field^34^ and solvated using the OPC^35^ water model with a minimum water buffer of 10 Å. For the *rigid* simulations, the protein structure was energy-minimized in the presence of the ligand, which was parametrized with the OpenFF Sage Force field 2.0.0.^36^

#### 2.1.2 Protein–Ligand Complex Minimization

For the *rigid* simulations, the protein configuration was first energetically minimized in the presence of the ligand. The complexes were first solvated in OPC water with a minimum buffer of 10 Å in tleap. The water was then minimized using 1500 steps of steepest descent with all heavy atoms of the ligand and protein restrained with a restraint weight of 100 kcal/mol/Å^2^. This was followed by a second energetic minimization of 1500 steps, in which only the backbone heavy atoms of the protein were restrained with the same force constant. The water and ligand were then removed, and the resulting protein configurations were used for the solvated protein minimizations for the *rigid* simulations. The remainder of the preparation steps (below) were identical for both the *rigid* and *flexible* simulations.

#### 2.1.3 Solvated Protein Simulation Energy Minimization

The proteins were first solvated in a box of aqueous OPC water with a minimum buffer of 10 Å in tleap. The systems were then energetically minimized using 1500 steps of steepest descent, with the water being unrestrained and all heavy atoms of the protein being restrained with a force constant of 100 kcal/mol/Å^2^. In the preparation of the *flexible* simulations, the systems were then energetically minimized using 1500 steps of steepest descent, with only the backbone heavy atoms of the protein being restrained with the same force constant.

#### 2.1.4 Equilibration

The energetically minimized systems were then equilibrated with molecular dynamics simulations. First, the systems were heated from 0 to 300 K over 240 ps at constant volume and temperature using a Langevin thermostat with a collision frequency of 2 ps^–1^.^37–39^ This was followed by a 10 ns MD simulation at a constant temperature of 300 K and pressure of 1 bar using the same thermostat and the position scaling barostat^40^ with a relaxation time of 0.5 ps^–1^, in which the protein atom restraints were decreased gradually from an initial value of 100 kcal/mol/Å^2^ to the production run restraint strength of 2.5 kcal/mol/Å^2^. This was followed by a second equilibration MD run of 10 ns at constant temperature and pressure, with 2.5 kcal/mol/Å^2^ restraints.

#### 2.1.5 MD Production Runs

The production MD runs were 100 ns at constant volume and a temperature of 300 K, regulated by a Langevin thermostat, with a time constant of 1 ps for the heat bath coupling and a collision frequency of 1 ps^–1^, and the first 20 ns were discarded and considered the final equilibration. All simulations were conducted under rectangular polyhedral periodic boundary conditions. The equilibration and production MD runs were performed using GPU-accelerated PMEMD,^41,42^ with a time step of 2 fs, and the SHAKE algorithm^43^ was used to constrain bond lengths involving hydrogens. Electrostatics were modeled with PME using a 10 Å direct space cutoff, and LJ interactions were computed up to 10 Å, with long-range effects corrected using the default isotropic method. Nonbonded parameters, including Lennard-Jones radii and well depths, were derived from the ff14SB force field. The protein and water configurations were output every 1 ps, yielding 80,000 frames.

### 2.2 GIST and SSTMap Solvation Mapping

GIST is a computational method that postprocesses MD trajectories to estimate and map solvation thermodynamic quantities onto a high-resolution grid. Thermodynamic quantities are estimated by using a spatial discretization of the equations of IST. In GIST, the energy density of a voxel, $E_{\mathrm{vox}}^{\mathrm{dens}}$ , is estimated from molecular dynamics trajectories by

$$E_{\mathrm{dens}}^{\mathrm{vox}}=\frac{\rho_{\mathrm{vox}}}{\rho^{{}^{\circ}}}\frac{1}{N_{\mathrm{vox}}}\sum_{i}^{N_{\mathrm{vox}}}(E_{i}-E_{\mathrm{neat}})$$ 1.1

where ρ_vox_ is the number density of the voxel, ρ° is the number density of the neat water system under the same thermodynamic conditions, *N*_vox_ is the number of water molecules found in the voxel over the course of the simulation, *E*_*i*_ is the potential energy of the water molecule interacting with the rest of the system, and *E*_neat_ is the average potential energy of a water molecule in the neat system. We refer the reader to Lazaridis,^44^ Nguyen et al.,^45^ and Ramsey et al.^46^ for extensive details on IST and GIST calculations.

For the *rigid* and *flexible* simulations of all 34 systems, GIST was applied to the 80 ns of the production runs using an in-house version of cpptraj GIST. The in-house version of cpptraj GIST varied from the public version in that it had additional code to map out hydrogen bond properties of water on the grid.

SSTMap utilizes a hydration site approach (HSA) to map out solvation and structural properties of water in high-density 1 Å radius spherical regions (hydration sites). Structural and thermodynamic quantities of water in each hydration site are calculated from an analysis of MD trajectories. Hydration site analyses using SSTMap were performed over the last 20 ns of the production runs.

#### 2.2.1 GIST Details

The grid dimensions were set to ensure that the GIST box included the whole system and were set independently for each simulated system. The grid spacing was 0.5 Å along each axis, yielding a voxel volume of 0.125 Å^3^. All default quantities were output, including the GIST-estimated total solvation energy, water–water and protein–water energies, and solvent densities in each voxel, as well as the corresponding per water quantities.

The total water energy values, *E*_tot_, are referenced to the neat OPC water energies (−12.259 kcal/molecule).

Error analysis on the GIST quantities used block averaging with the 80 ns production runs separated into four 20 ns blocks.

#### 2.2.2 Binding Cavity Subvolumes and Integrated Energetic and Structural Quantities

The binding cavity subvolumes were defined by the set of voxels, whose center was within a specified distance of any heavy atom of the system’s cognate ligand. The MD simulations were run without the ligands present; hence, the volume is with reference to the aligned cocrystal ligand coordinates. The properties of water were calculated in subvolumes ranging from 3 to 10 Å from the cognate ligand, with increments of 0.5 Å (Figure 2). GIST postprocessing (GISTPP)^6^ was used to define the subvolumes and calculate the thermodynamic properties of water within each subvolume.

**Figure 2:**
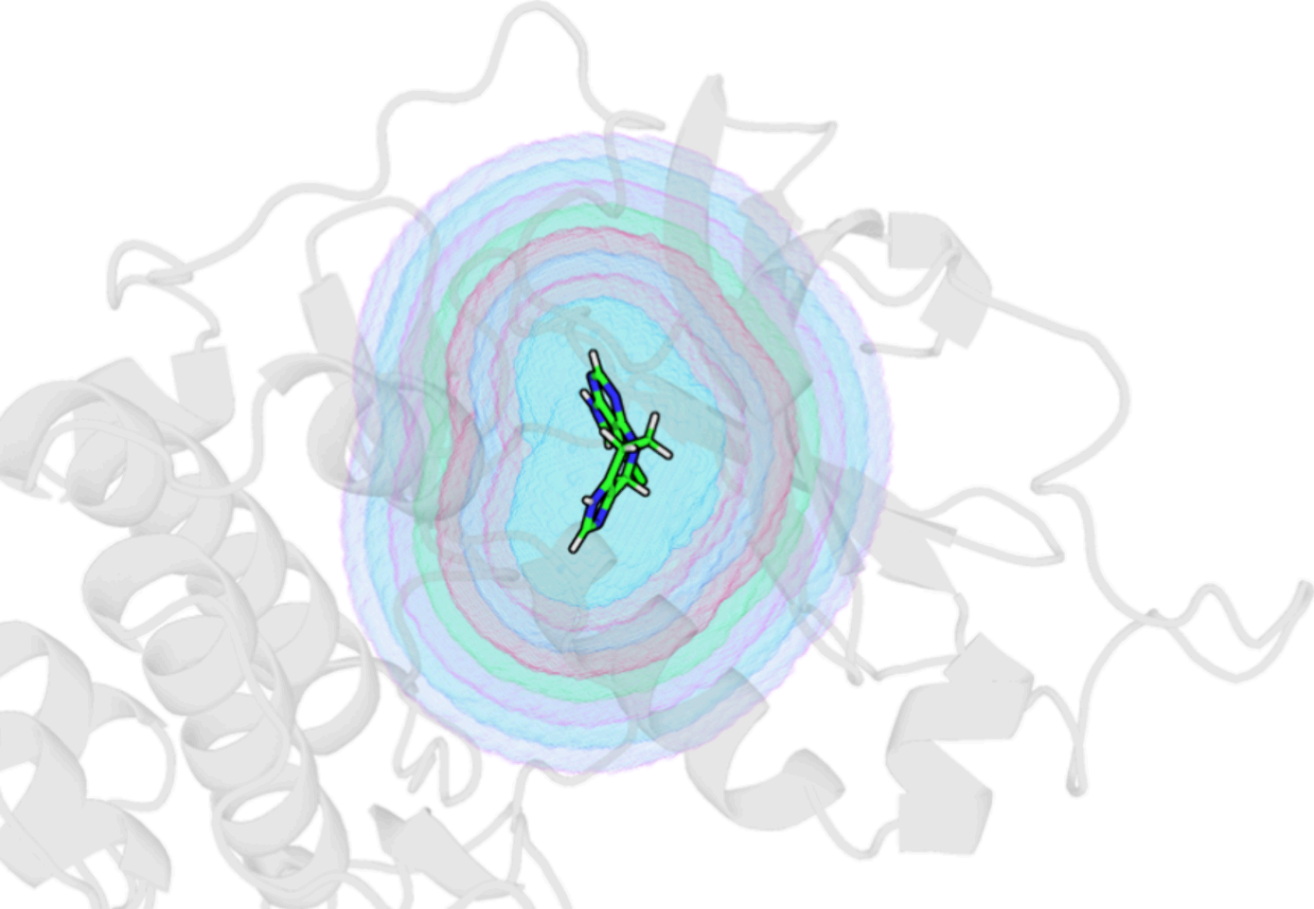
Depiction of binding site subvolumes about the cognate ligand. Though the ligand is not present in the simulations, the subvolumes are defined by the regions within a certain distance of any heavy atom of the aligned cocrystal ligand coordinates. The inner blue region is the smallest subvolume defined by all GIST voxels, whose center is within 3 Å of the ligand. Each differently shaded region here is demarcated at 1 Å intervals. The full shaded region is the largest subvolume, which includes all voxels within 10 Å of the ligand. The integrated quantities are the sums of all voxel quantities over the region.

The total values for the GIST quantities in a subvolume are simply the sum of the voxel density quantities for all voxels in the subvolume. We refer to these values collectively as *integrated* values. The per water quantity is simply this value divided by the number of water molecules.

The upper limit of 10 Å was chosen based on our prior study (Chen et al.), which showed that integrating the solvation energy over this subvolume was sufficient to closely estimate the total solvation energy of small molecules.^47^ Based on this, we use the energetics of water in a 10 Å subvolume around the cognate ligand as an estimate of the full solvation energy of the binding site as it largely encompasses both the region from which solvent is displaced and the region in which water restructures itself around a complex, respectively, referred to as *V*_disp_ and *V*_rest_ by Gilson and Kurtzman.^10^ We refer to the 10 Å subvolume as *V*_cav_.

The lower limit of 3 Å from any ligand heavy atom for the subvolume was chosen because it has often been used to approximate the volume from which water is displaced upon ligand binding.^4,48,49^ We refer to this volume as *V*_disp_.

#### 2.2.3 Solvent-Accessible Surface Area

GISTPP was used to generate the solvent-accessible surface areas by using the default density inputs.

#### 2.2.4 Geometric Definition of Hydrogen Bonds and Neighbors

We use a geometric definition of a hydrogen bond in which a noncovalent polar interaction is considered a hydrogen bond when the distance between the two heavy atoms is less than 3.6 Å and the angle of acceptor–donor–hydrogen is less than 30°.^50^ Our in-house version of cpptraj GIST mapped out the densities of water–protein and water–water hydrogen bonds on the GIST grid. Water molecules or heavy atoms are considered to be “neighbors” if they are within 3.6 Å of each other.

#### 2.2.5 Hydration Site Analyses

SSTMap with default settings was applied to the last 20 ns of the *rigid* and *flexible* simulations for all 34 systems investigated. Hydration sites were characterized as either acceptors or donors if 60% or more of the water molecules found in the hydration site made the appropriate acceptor or donor interaction. If both values were above 60%, then the hydration site was characterized as both donor and acceptor.

## 3 Results

### 3.1 Results Overview

In this study, we apply GIST and SSTMap analysis tools to characterize the structure and energetics of water solvating 34 *rigid* and *flexible* protein binding cavities. As detailed in Section 2, the term “*rigid”* refers to simulations of systems, in which all heavy atoms are restrained about the positions in the ligand-bound crystal structure and the term “*flexible”* refers to results from simulations for systems, in which the side chain heavy atoms are left unrestrained and are free to move away from their ligand-bound configuration.

Our fundamental finding is that binding cavity solvation energies are significantly less favorable in *rigid* systems than in *flexible* systems. This was true for 33 out of the 34 systems studied, and on average, the significant energy difference in the binding site cavities (*V*_cav_) was 14.4 kcal/mol. We can understand these differences in energies from our analysis of water–protein hydrogen bonds, where we find that in the *flexible* systems, water forms 8.5 more hydrogen bonds with the protein surface on average than in the *rigid* systems. We find, however, that these improved water–protein interactions come at a cost. In the formation of more favorable interactions with the protein, water sacrifices some of its favorable interactions with neighboring water molecules. These opposing contributions together yield the observed net favorability of the solvation energy for *flexible* over *rigid* binding sites of 14.4 kcal/mol. This finding motivates us to propose a conceptual extension to the traditional principle of protein–ligand complementarity. In the presence of a ligand, the protein adopts conformations that optimize hydrogen bonds or make hydrophobic contacts with the ligand, while in the absence of the ligand, the protein relaxes into conformations that are complementary to water.

This substantial difference in solvation energy led us to hypothesize that, at least for some systems, there may be little overlap in the binding cavity configurations sampled when the ligand is absent (*flexible*) compared to those sampled when the ligand is present (*rigid*). To explore this, we closely investigated the solvation and side chain configurations in two binding cavities that were highly enclosed and capable of binding bidentate ligands, topographies that had been previously identified as difficult to solvate.^11,51^ For comparison, we also investigated a system that was bound to a bidentate ligand but was not highly enclosed (PDB ids: 2GTK, 3BIZ, and 3CCW). Here, we find that there are side chains in the unligated *flexible* systems that never or rarely sample configurations that would make the appropriate hydrogen bond contacts with the ligand if it were present in its cognate conformation. This is consistent with the concept of “induced-fit,” which suggests that a binding cavity undergoes a conformational change in order to accommodate a ligand.

The structure of the results section is as follows: In Section 3.2, we discuss the differences in the *flexible and rigid* binding cavity volumes and how the change in volume and number of water molecules affects the analysis of binding site solvation energetics and hydrogen bonding. In Section 3.3, we present an analysis of the total solvation energetics of the *rigid* and *flexible* cavities on average and how this quantity varies for each protein system. In Section 3.4, we deconstruct the solvation energetics into contributions from protein–water and water–water interactions and contextualize these energies by assessing the hydrogen bonding characteristics of water solvating the binding cavity. Finally, in Section 3.5, we provide a detailed analysis of solvation energetics and water structures in three systems (PPAR-γ, HMDH, and WEE1). Two of these systems (PPAR-γ and HMDH) bind bidentate ligands within enclosed binding cavities, a topography previously identified as challenging to optimally solvate.^11^ In these systems, water is unable to form the same complementary hydrogen bonds with the protein that the cognate ligand makes. We show that in the *flexible* simulations, side chains restructure away from their cognate conformations to adopt geometries that are more complementary to the solvent, thereby lowering solvation energetics. Notably, some of these side chains rarely or never sample conformations required to be complementary to the cognate ligand. As a point of contrast, we also analyze WEE1, which binds a bidentate ligand in a more open binding site. In this cavity, water makes hydrogen bond interactions comparable to those of the cognate ligand in the *rigid* cavity, and the side chains in the flexible simulation largely preserve conformations compatible with ligand binding. This suggests that binding cavity enclosure impacts solvation and, in conjunction, configurational sampling.

### 3.2 Cavity Size

In this section, we use the average number of water molecules in *V*_cav_ as a proxy for the effective cavity volume (Figures 3 and S1). This volume differs between the *flexible* and *rigid* systems, as the movement of the side chain effectively opens, allowing more water molecules into the cavity, or partially closes, effectively displacing water molecules from the cavity. For the 34 systems studied, we observed that the binding cavity of 24 of the systems opened to accommodate more water and 10 of the systems closed to accommodate fewer water molecules. There was a significant variation in the magnitude of opening or closing (Figure 3), and we found no correlation between the cavity opening or closing and the energy of water in the rigid cavity (*R* ^2^ = 0.21) or the changes in the energy between the *rigid* and *flexible* systems (*R* ^2^ = 0.001).

**Figure 3:**
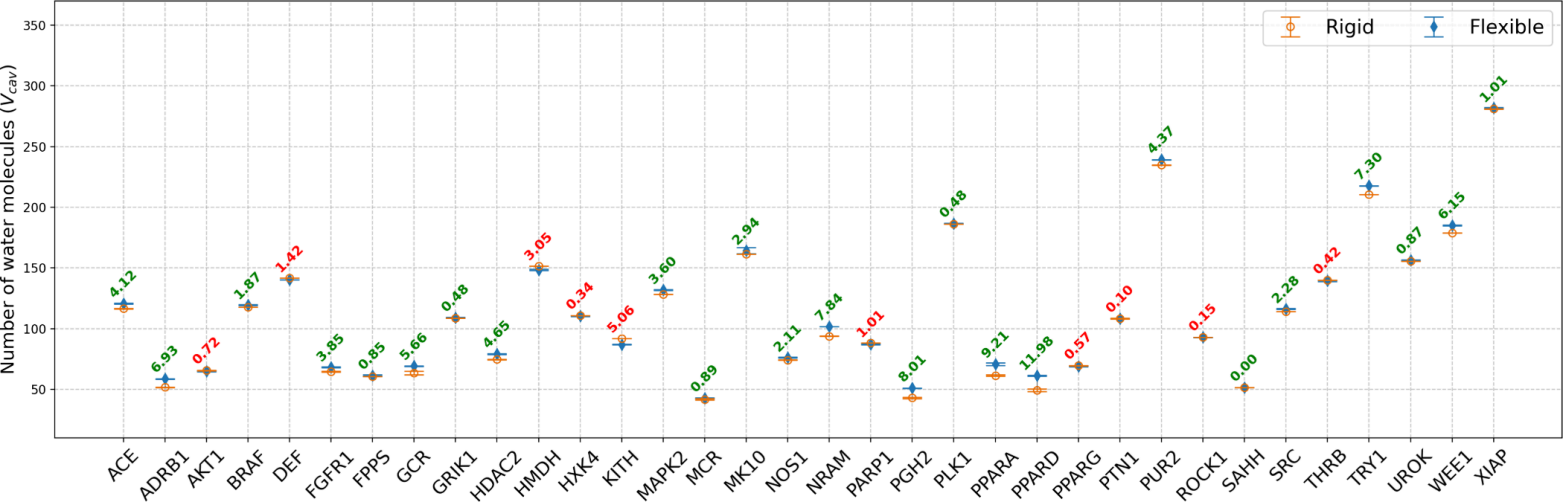
Number of water molecules in *V*_cav_ for the *rigid* and *flexible* binding pockets for all 34 systems. Data from the *rigid* simulations are hollow orange circles, and *flexible* simulation data are represented as solid blue diamonds. The text shows the difference between *rigid* and *flexible*, with red text denoting that the flexible cavities have fewer water molecules and green text denoting that the rigid cavities have fewer. Data for other subvolumes (3.0–9.5 Å) are reported in the Supporting Information (Figures S1 and S2).

Direct comparisons of the total energetic properties of water in the *rigid* and *flexible* binding cavities are complicated by the fact that they involve comparisons of different numbers of water molecules. For this reason, we report both the per water quantities and the total integrated solvation energy for each cavity subvolume.

Regardless of whether the cavities opened or closed, the energy per water for all of the *flexible* systems was more favorable than for the *rigid* systems (Figure 5).

### 3.3 Solvation Energetics in the Binding Cavity

We find that, on average, the GIST estimated energy is significantly more favorable for the *flexible* systems than for the *rigid* systems (Figure 4). For the 10 Å subvolume of the binding cavity, the GIST estimated difference in average energy is 14.43 kcal/mol. On a per water molecule basis, the average difference in solvent energy is 0.17 kcal/mol, with the *flexible* systems being more favorable.

**Figure 4:**
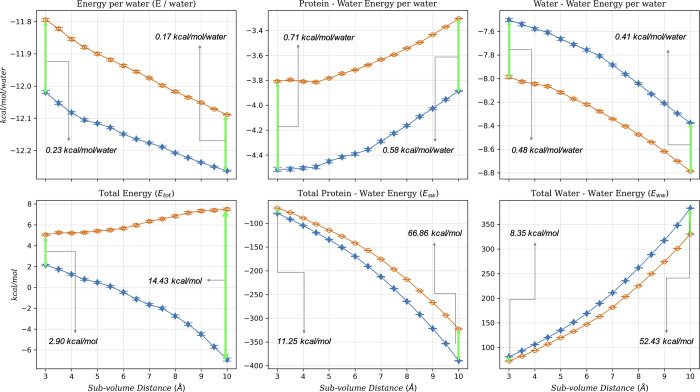
Total solvent energy (*E*_tot_), protein–water interaction energy (*E*_sw_), and water–water interaction energy (*E*_ww_) per water values (*y*-axes) averaged over the 34 systems within each subvolume (*x*-axes). Data from the *rigid* simulations are hollow orange circles, and *flexible* simulation data are represented as solid blue diamonds. The ‘total’ quantities are referenced to the energy of the same number of water molecules as found in the appropriate subvolume in a neat system under the same thermodynamic conditions. The error bars are the error in the mean.

The average total energy and energy per water for the *V*_disp_ and *V*_cav_ regions are summarized in Table 1. The average energy of water molecules in the binding cavity is more favorable in the *flexible* than in the *rigid* cavities, and this trend holds for every one of the 34 systems (Figure 5) that we investigated, however, the range of per water energy differences is from almost negligible (0.02 kcal/mol for XIAP, PDB id: 3HL5 ^52^) to considerable (0.58 kcal/mol for SAHH, PDB id: 1LI4 ^53^). The total solvent energy in the binding cavity volume (V_cav_) is more favorable in the *flexible* cavities for 33 of the 34 systems with peroxisome proliferator-activated receptor delta (PPAR-δ, PDB id: 2ZNP^54^) being an outlier, and the total solvent energy has a considerable range from −3.82 kcal/mol for PPAR-δ to 47.00 kcal/mol for the angiotensin-converting enzyme (ACE, PDB id: 3BKL^55^). We note that for PPAR-δ, the *flexible* cavity (*V*_cav_) has 11.98 more water molecules on average than the *rigid* cavity and, as the water molecules on average in both systems are unfavorable compared to neat water (to which the energies are referenced), this leads to the total solvent energy being less favorable in the *flexible* system compared to the *rigid*.

**Table 1:** Average Total Solvation Energy (*E*_tot_) and Energy Per Water (*E*/Water) for the 34 Systems in Subvolumes *V*_disp_ (3 Å) and *V*_cav_ (10 Å)^a^

	*E*_tot_			*E*/water		
volume	*rigid*	*flexible*	Δ*E*_total_ (*rigid–flexible*)	*rigid*	*flexible*	Δ*E*/water (*rigid–flexible*)
*V*_cav_	7.51	–6.92	14.43	–12.09	–12.26	0.17
*V*_disp_	5.05	2.14	2.90	–11.79	–12.02	0.23

a The total energy is referenced to neat water (kcal/mol). The ‘total’ quantities are referenced to the energy of the same number of water molecules as found in the appropriate subvolume in a neat system under the same thermodynamic conditions.

**Figure 5:**
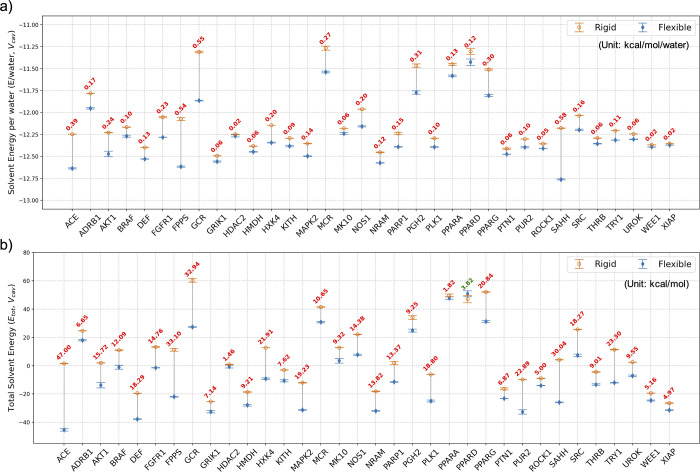
Solvent energy (a) per water molecule and (b) total values for the *rigid* and *flexible* binding pockets in *V*_cav_ (10 Å subvolume) for 34 systems. Data from the *rigid* simulations are hollow orange circles, and *flexible* simulation data are represented as solid blue diamonds. Differences between the values of *rigid* and *flexible* pockets for each system are indicated in red text. The total energy values are referenced to the energy of the same number of neat water molecules as those found in *V*_cav_. Data for other subvolumes (3.0–9.5 Å) are reported in the Supporting Information (Figures S3 and S4).

Although our analysis is focused on the 10 Å subvolume (*V*_cav_), the average values in the smaller volume (*V*_disp_, 3 Å subvolume), which refers to the solvent displacement region, are also significantly different for the *flexible* and *rigid* systems. In this region (*V*_disp_), the average differences in the total energy and energy per water between two conformations are 2.90 and 0.23 kcal/mol, respectively.

### 3.4 Protein–Water and Water–Water Interaction Energy and Hydrogen Bonds

The total solvation energy can be broken down into a sum of two terms: one from the contribution of protein–water (*E*_sw_) interaction and another from the water–water (*E*_ww_) interaction. Figure 6 shows that, while the total water energy is more favorable for the *flexible* systems, this total energy difference has two opposing contributions: a protein–water contribution that is more favorable for *flexible* systems and a water–water contribution that is less favorable for *flexible* systems. Figures 7 and 8 show that this trend holds for all 34 systems of the systems investigated in this study, with the magnitude of the protein–water differences being larger than the water–water difference for all systems investigated.

**Figure 6:**
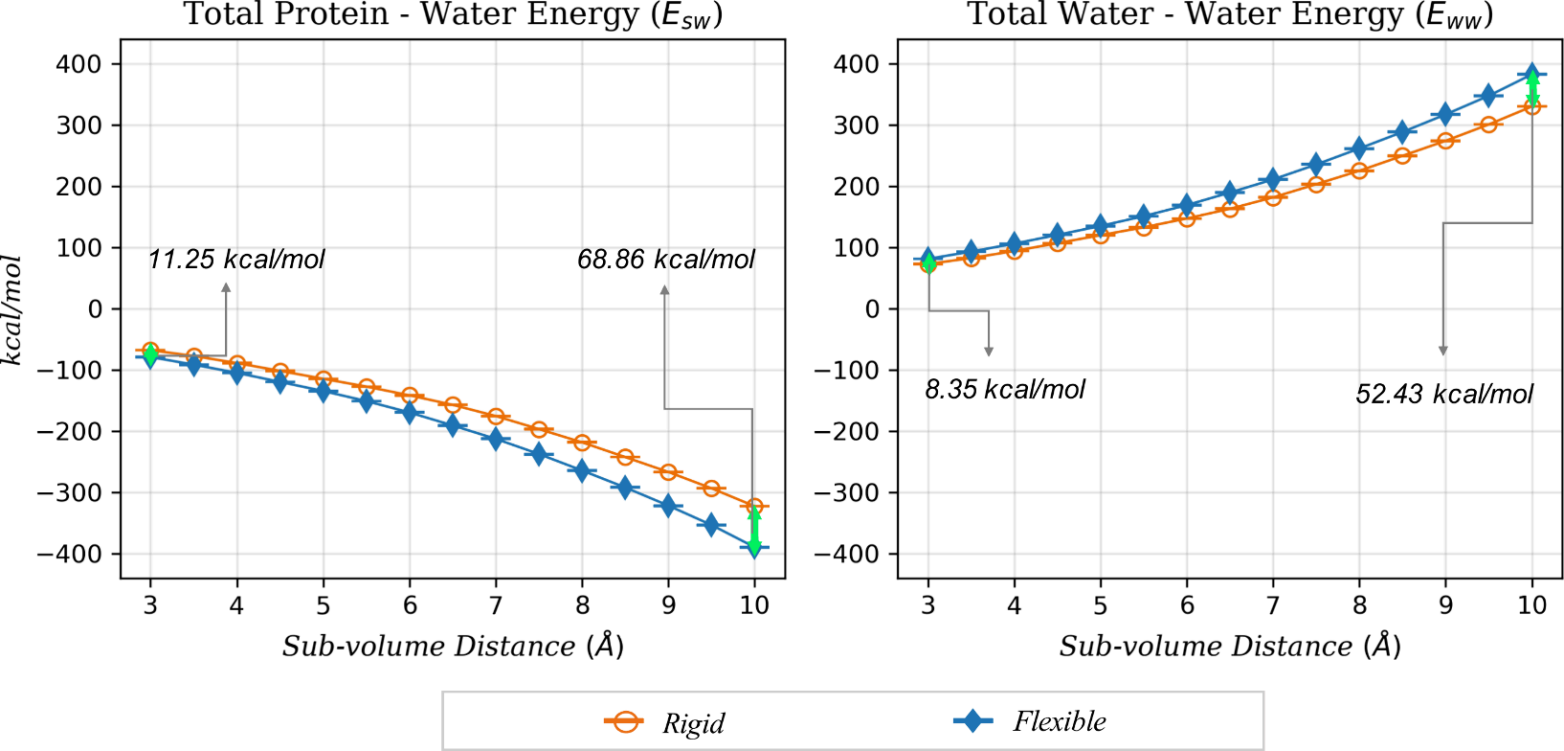
Protein–water and water–water energies averaged over 34 systems studied within each subvolume (*x*-axis). Data from the *rigid* simulations are hollow orange circles, and *flexible* simulation data are represented as solid blue diamonds. The water–water energy is referenced to the average water–water energy per water in a neat system. This data is also shown in Figure 4 but reproduced here for convenience. The total energy values are referenced to the energy of the same number of neat water molecules as found in each subvolume.

**Figure 7:**
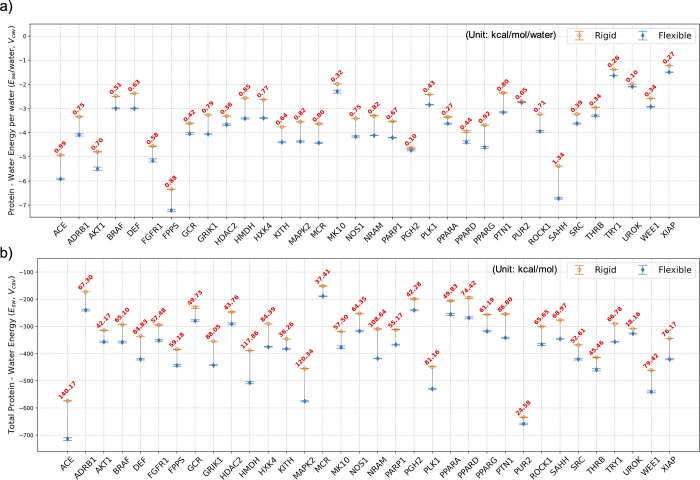
Protein–water interaction energies (a) per water molecule and (b) total values in the *rigid* and *flexible* binding pockets (*V*_cav_) for all 34 systems. Data from the *rigid* simulations are hollow orange circles, and *flexible* simulation data are represented as solid blue diamonds. Differences between the two values are in the text, with red text denoting that the *flexible* cavities have lower energies. The total energy values are referenced to the energy of the same number of neat water molecules as found in *V*_cav_. Data for the other subvolumes (3–9.5 Å) can be found in the Supporting Information (Figures S5 and S6).

**Figure 8:**
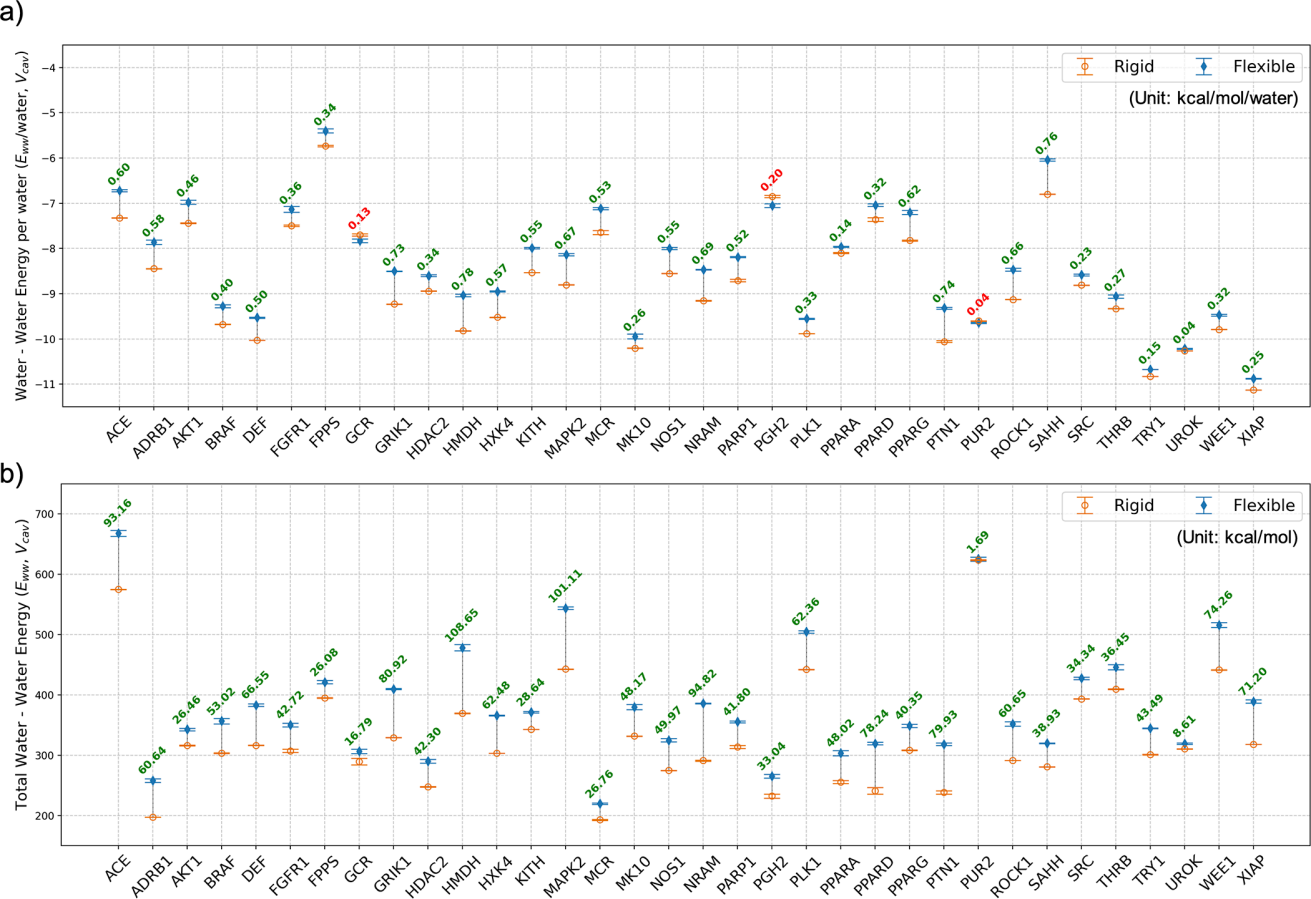
Water–water interaction energy (a) per water molecule and (b) total values for the *rigid* and *flexible* binding pockets (*V*_cav_) for all 34 systems. Data from the *rigid* simulations are hollow orange circles, and *flexible* simulation data are represented as solid blue diamonds. Differences between the values for the *rigid* and *flexible* pockets are in the figure, with green text denoting that the water–water energy is more favorable in the *rigid* cavity and red text denoting that the water–water energy in the *flexible* cavity is more favorable. Data for other subvolumes are reported in the Supporting Information (Figures S7 and S8).

The more favorable protein–water energy in the *flexible* systems can be understood in part by the differences in protein–water hydrogen bonds observed in our simulations. Figure 9 shows that, on average, water forms 8.51 more hydrogen bonds with the protein in *V*_cav_ of the *flexible* systems than in the *rigid* systems. Figure 10 shows that in every *flexible* system, water forms more hydrogen bonds with the protein than in the *rigid* systems, and that the differences in the number of protein–water hydrogen bonds range from a difference of 2.78 hydrogen bonds for UROK (PDB id: 1SQT^56^) to 18.46 hydrogen bonds for HMDH (PDB id: 3CCW). We also investigated whether the difference in the number of protein–water hydrogen bonds between the *rigid* and *flexible* systems could be related to the differences in the number of water molecules observed in the binding cavities. We found that regardless of whether there were greater or fewer water molecules in the binding cavity of the *flexible* systems, the water formed more protein–water hydrogen bonds than those with the *flexible* systems. Additionally, the difference in the number of water molecules in the binding cavity between the *rigid* and *flexible* systems was uncorrelated with the difference in the number of protein–water hydrogen bonds (*R* ^2^ = 0.00008).

**Figure 9:**
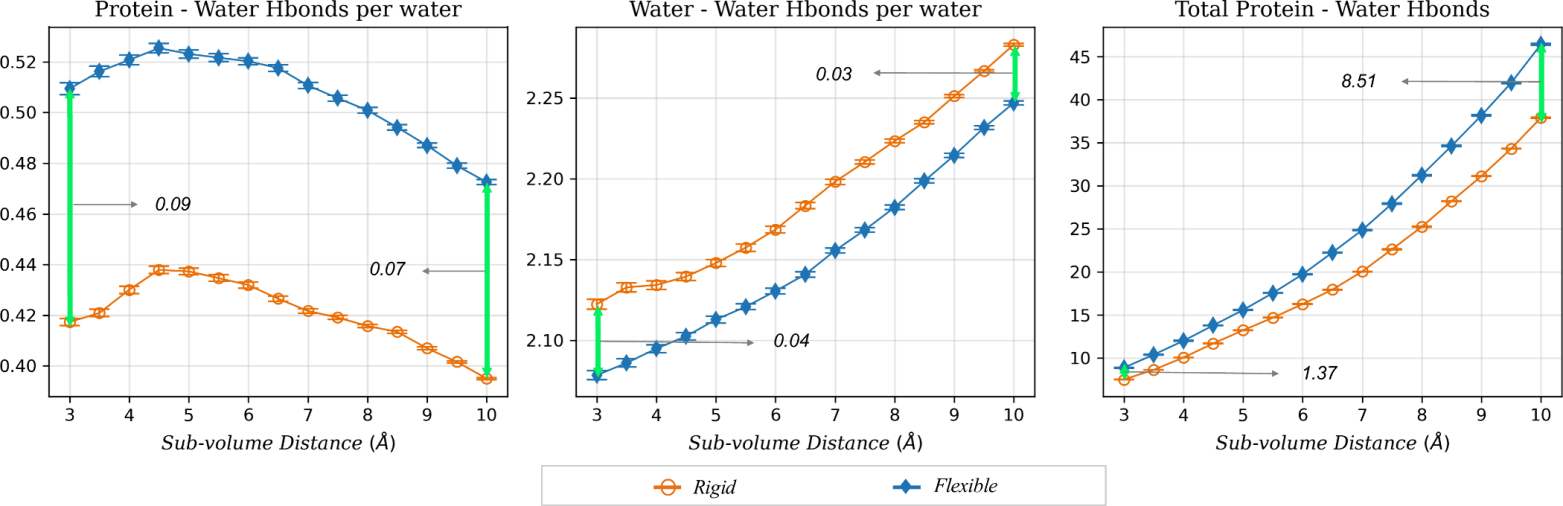
Number of protein–water, water–water per water molecule, and total protein–water hydrogen bonds for the *rigid* and *flexible* binding cavities in each subvolume averaged over all 34 systems. Data from the *rigid* simulations are hollow orange circles, and *flexible* simulation data are represented as solid blue diamonds.

**Figure 10:**
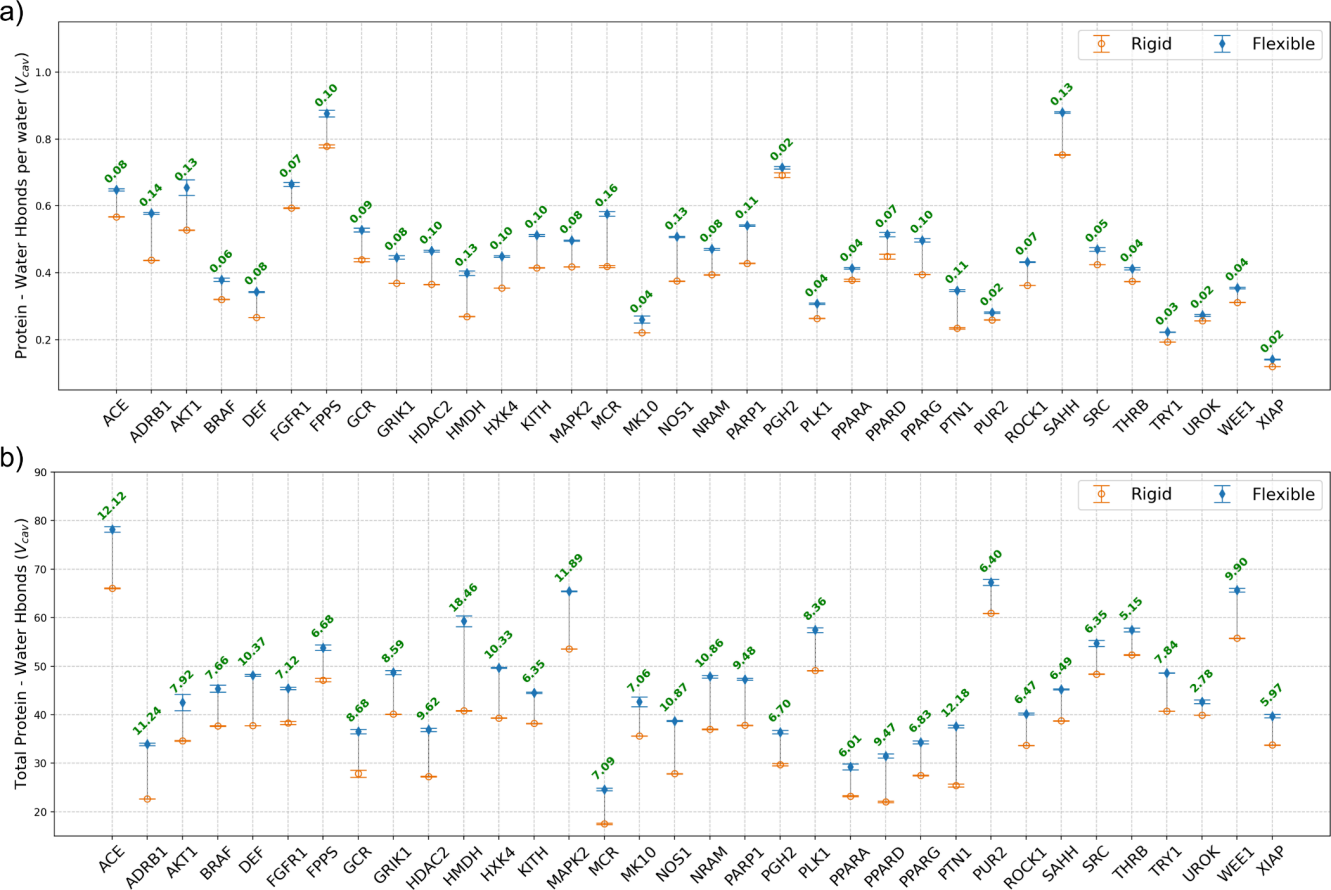
Average number of protein–water hydrogen bonds (a) per water molecule and (b) total values for the *rigid* and *flexible* binding pockets (*V*_cav_) for all 34 systems. Data from the *rigid* simulations are hollow orange circles, and *flexible* simulation data are represented as solid blue diamonds. Differences between the values of *rigid and flexible* pockets for each system are shown in green when the *flexible* value is higher and in red when the *rigid* value is higher. Data for other subvolumes are reported in the Supporting Information (Figures S9 and S10), while water–water hydrogen bonds are illustrated in Figures S11 and S12.

A pattern of protein and water restructuring from the *rigid* to *flexible* systems emerges from this data. In the *rigid* systems, the protein side chains adopt conformations that are suitably complementary to the ligand; however, water does not make fully complementary hydrogen-bonding interactions with the proteins when they are in these conformations. When the restraints on the side chain motions are removed, the protein and water restructure in a manner that lowers the energy of the binding site water and allows the formation of more complementary protein–water interactions, as illustrated by the protein–water hydrogen bond data (Figures 9 and 10). In turn, in the *rigid* systems, water is unable to make as many favorable interactions with the protein and instead, following Le Chatelier’s principle, prioritizes forming more favorable interactions with the neighboring water molecules, as illustrated by the lower solvation energy and water–water energy for *rigid* systems (Figure 8). Conversely, while the *flexible* systems restructure to allow more favorable protein–water interactions, this comes at the cost of frustrating the water–water interactions in the *flexible* systems compared to those in the *rigid* systems.

### 3.5 Systems Bound to Multidentate Cognate Ligands

In a previous study, we investigated the structural frustration of water molecules solvating *rigid* binding sites.^11^ Inspired by the Frank and Evans iceberg model of hydrophobic hydration,^57^ we characterized *optimal* hydration as water molecules’ ability to pack into a binding cavity in such a way as to make favorable hydrogen-bonding interactions with both the protein surface and water neighbors. Conversely, we characterized *suboptimal* hydration as situations in which water is unable to simultaneously form favorable hydrogen bonds with both its neighboring water molecules and the protein surface.

In particular, we investigated the water molecules solvating the residues that form hydrogen bonds with aspartate in the *rigid* binding site of the aspartyl-specific protease Caspase-3,^58^ a highly enclosed binding pocket. In this cavity, the structure of the water was *frustrated* in that the water in the cavity was unable to form the same number of hydrogen bonds with the protein surface as the carboxylate of the ligand, and the water–water interactions were unfavorable compared to those formed in bulk water. Our physical interpretation behind this had to do with how water molecules pack in the liquid phase. In neat water, the oxygens of two water molecules do not approach a distance closer than 2.4 Å, as the radial distribution function is effectively zero until this distance, and the optimal (most probable) distance between two water molecules is 2.8 Å. Many functional groups in pharmaceutical compounds, such as the aspartyl carboxylate in this prior study, have donor–acceptor heavy atom pairs that are less than 2.4 Å from each other (Figure S13). Water, due to its inability to pack so closely together, is unable to place the donors and acceptors at the same positions as the ligands in these cases.

Here, we describe in greater detail the binding site solvation of several systems (PPAR-γ, HMDH, and WEE1) proximal to the region where the bidentate ligands form hydrogen bond contacts with the protein surface. For each functional group of the protein that forms hydrogen bonds with the bidentate ligand, we detail metrics of solvation, including the number of water neighbors (a metric of solvent exposure), how many hydrogen bonds they are able to make with the solvent, and how these values differ in the *rigid* and *flexible* simulations. Our analysis focuses on PPAR-γ (peroxisome proliferator-activated receptor gamma, PDB id: 2GTK), HMDH (HMG-CoA reductase, PDB id: 3CCW), and WEE1 (serine/threonine-protein kinase WEE1, PDB id: 3BIZ), each of which has a bidentate ligand but has binding site topographies that differ considerably.

#### 3.5.1 System 1: PPAR-γ (PDB ID: 2GTK)

The binding site of PPAR-γ tightly encloses ligand 208[Fn fn1]’s carboxylate, which accepts a total of four hydrogen bonds with protein residues HIS-449, TYR-473, SER-289, and HIS-323 (Figure 11, left and Figure 12). While the binding cavities in both the *rigid* and *flexible* simulations are large enough to accommodate a carboxylate (Figure 11, middle and right), neither is large enough to accommodate two water molecules. Hence, when these cavities are solvated by one water molecule, it accepts fewer hydrogen bonds than the carboxylate group of the ligand. This is due to the limited ability of water to pack into tightly enclosed areas. The distance between the two carboxylate oxygens is about 2.3 Å, which is closer than two water oxygens approach due to their van der Waals radii (e.g., the radial distribution of water is effectively zero at this distance), and the binding cavity in neither the *flexible* nor *rigid* simulations is large enough to accommodate two water molecules.

**Figure 11:**
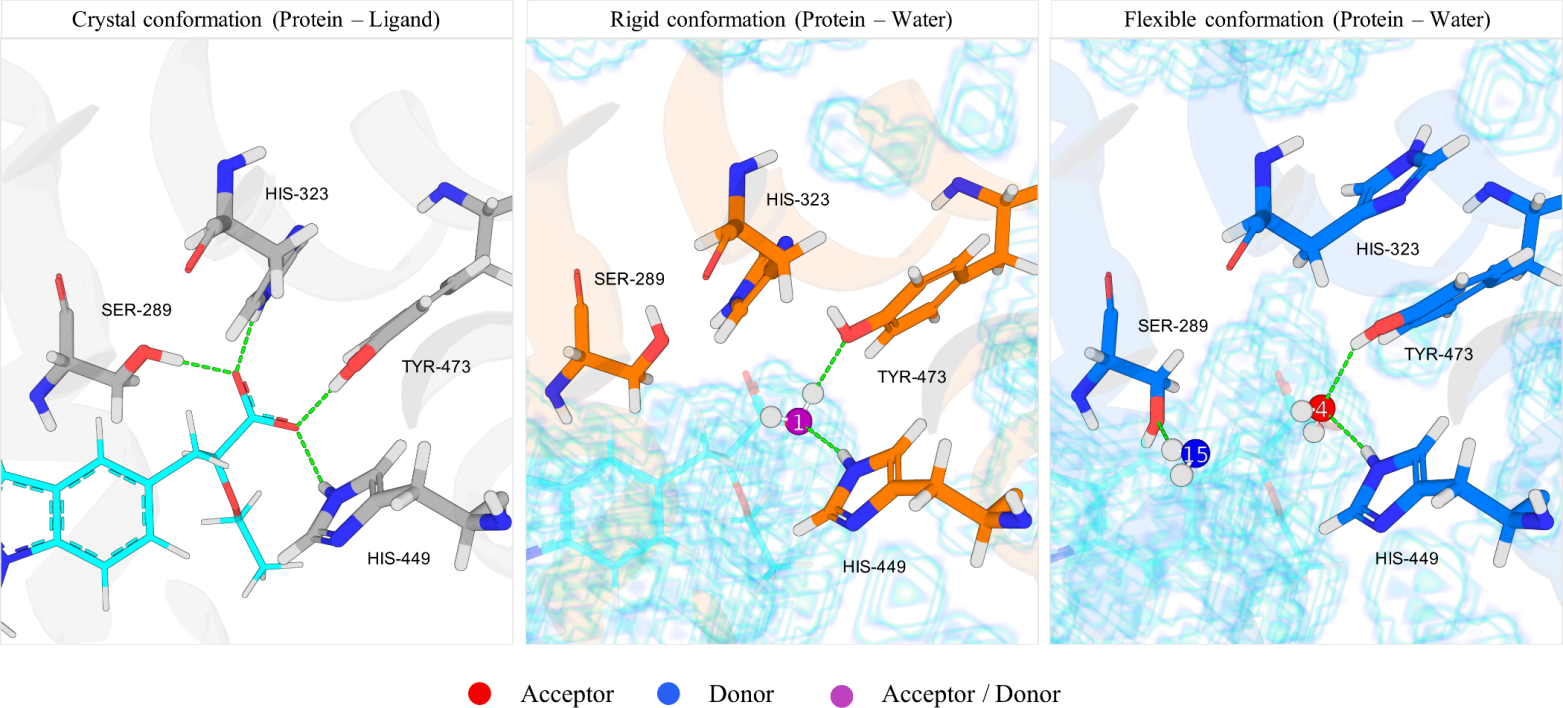
Binding cavity of PPAR**-**γ (PDB id: 2GTK). (Left) Ligand 208 and the hydrogen bonds that its carboxylate forms with the protein. (Middle) Solvent-accessible surface (scaffolded) and SSTMap hydration site that interacts comparably to the ligand carboxylate in the *rigid* simulation. (Right) Same results for the *flexible* simulation. Hydration sites that donate hydrogen bonds to the surface are colored in blue. Those that accept are in red, and those that both donate and accept are colored in purple.

**Figure 12:**
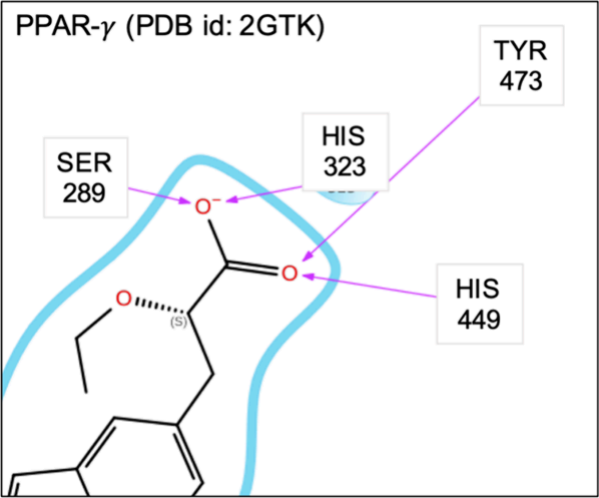
Two-dimensional (2D) diagrams of the interactions between ligand 208 and the binding site of PPAR-γ (2GTK). Only the side chains and interactions relevant to the analysis are shown. This diagram was generated using Schrödinger Maestro. Full 2D diagram is provided in Figure S14a of the Supporting Information.

In both the *rigid* and *flexible* systems, a hydration site is placed to form hydrogen bonds with HIS-449 and TYR-473 and form comparable contacts with the protein that the ligand carboxylate makes (Figure 11, middle and right). This leaves the H-bond contacts with SER-289 and HIS-323 unsatisfied in the *rigid* structure when the ligand is not present. In the *rigid* simulations, the hydroxy groups of SER-289 and HIS-323 are poorly solvated with few water neighbors, and almost no hydrogen bonds are formed with water (Tables 2 and 3).

**Table 2:** Average Number of Water Molecule Neighbors (within 3.5 Å) of the Functional Groups of Side Chains That Formed Hydrogen Bonds with the Ligand in the Cognate Conformation of PPAR**-**γ (PDB ID: 2GTK)­^a^

	SER289-OG	HIS323-NE2	TYR473-OH	HIS449-NE2	total H_2_O neighbors
*rigid*	0.30	0.43	0.88	1.11	2.73
*flexible*	1.73	0.00	2.18	1.30	5.21

a This is a metric of how well solvated each functional group is.

**Table 3:** Average Number of Hydrogen Bonds Formed between Water and the Functional Groups of the Side Chains That Formed Hydrogen Bonds with the Ligand in the Cognate Conformation of PPAR**-**γ (PDB ID: 2GTK)

	SER289-OG	HIS323-NE2	TYR473-OH	HIS449-NE2	total H-bonds
*rigid*	0.09	0.00	0.73	0.80	1.62
*flexible*	1.23	0	1.78	0.83	3.84

In the *flexible* simulations, the protein, correspondingly, restructures to a configuration that is more *complementary* to the water. In the *flexible* simulations, the imidazole ring of HIS-323 flips to form an internal hydrogen bond with LYS-319 (not shown) and is no longer solvent-accessible, with no water neighbors and correspondingly no hydrogen bond contacts with water. The hydroxy of SER-289 moves to become more solvent-accessible and thus forms better hydrogen bonds with water. Overall, the serine hydroxy is significantly better hydrated in the *flexible* system with more neighbors (1.4) and more hydrogen bonds (1.14) than in the *rigid* simulation (Tables 2 and 3).

The reorganization of the protein in the *flexible* simulations has repercussions with regard to identifying ligands that are complementary to the protein surface. In the cognate structure, the ε-amino of HIS-323 is on the surface and can hydrogen bond with a complementary ligand, whereas in the predominant *flexible* structure, this hydrogen bonding site is not available to bind to the ligand. In both the cognate structure and the predominant *flexible* structure, the hydroxy of SER-289 is solvent-accessible; however, in the flexible system, the hydrogen bonding position has moved by 2.5 Å. We note that in the *flexible* systems, the side chains can move back and forth. In the *flexible* simulation, the hydroxy of SER-289 was proximal to the shown position (Figure 11, right) in 93.6% of the sampled frames and closer to the cognate structure in 6.4% of the sampled frames.

As water structures itself differently in the *rigid* and *flexible* binding cavities, it is difficult to compare hydration sites between them, as they often change positions. However, hydration site 1 (HS1) in the *rigid* system and hydration site 4 (HS4) in the *flexible* system both bridge hydrogen bonds between the same functional groups of TYR-473 and HIS-449, which makes the comparison more reasonable. The water in the *flexible* system has slightly more water neighbors and forms, on average, slightly more hydrogen bonds (0.28) with its water neighbors. In turn, it makes slightly fewer hydrogen bonds with the protein and overall makes slightly less favorable energetic interactions with the protein; however, the significantly better energetic interactions with its water neighbors compensate for this, and the energy of the hydration site is more favorable in the flexible system (Table 4).

**Table 4:** Protein–Water Interaction Energy (*E*_sw_), Water–Water Interaction Energy (*E*_ww_), and Total Energy (*E*_tot_) of Hydration Site 1 (HS1, *Rigid*) and Hydration Site 4 (HS4, *Flexible*)­^a^

	*E*_sw_	*E*_ww_	*E*_tot_
HS1 (*rigid*)	–5.82677	–4.83525	–10.662
HS4 (*flexible*)	–5.5404	–6.08115	–11.6216

a The total energy values are referenced to the energy of the same number of neat water molecules as those found in the hydration site.

#### 3.5.2 System 2: WEE1 (PDB ID: 3BIZ)

In contrast to PPAR**-**γ, the binding site of WEE1 does not tightly enclose the tridentate succinimide group of ligand 61E[Fn fn2] (Figures 13 and 14), and water is fully able to make all three hydrogen bonds with the protein in both the *rigid* and *flexible* simulations. While the water molecules cannot pack into the cavity to make these contacts from the same positions as the ligand, they are able to spread themselves out within the open cavity to make complementary hydrogen bond contacts with the protein. The distance between the nitrogen and oxygens in succinimide is 2.3 Å, a distance that is energetically prohibitive for two water molecules to have. In contrast, the water molecules that make the same contacts as the NH and carbonyl groups are 4.3 Å (HS0 and HS6) in the *rigid* system and 6.6 and 6.9 Å in the *flexible* system (HS2 with HS4 and HS19, respectively). Despite this, there is a reorganization of the protein in the *flexible* simulations such that the amide group of ASN-376 flips (in 88.1% of the simulation frames) so that the carbonyl is solvent exposed instead of the amino group (Table 5). This amide flipping also suggests that ligands that can donate or accept at that position will be complementary to the protein. The solvent exposure of the other protein functional groups that interact with the ligand remains relatively unchanged when comparing the *rigid* and *flexible* systems as does the number of hydrogen bonds (Table 6). Overall, the solvent reorganization energy of WEE1 is small compared to PPAR-γ, with the *flexible* systems being more favorable at 5.16 kcal/mol (*V*_cav_) compared to 20.84 kcal/mol for PPAR-γ.

**Figure 13:**
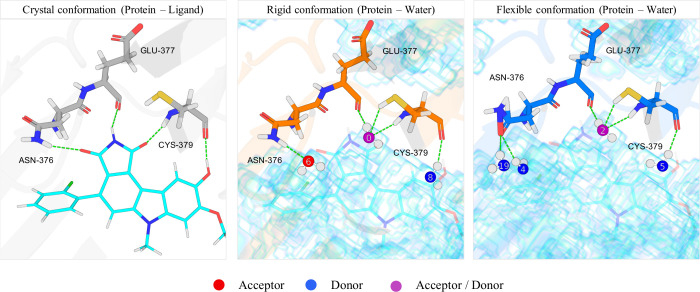
Binding cavity of WEE1. (Left) Ligand 61E and the hydrogen bonds that it forms with the protein. (Middle) Solvent-accessible surface (scaffolded) and SSTMap hydration sites that interact comparably to the ligand in the *rigid* simulation. (Right) Same results for the *flexible* simulation with the time averaged structure of the protein. Hydration sites that donate hydrogen bonds to the surface are colored in blue. Those that accept are in red and those that both donate and accept are colored in purple.

**Figure 14:**
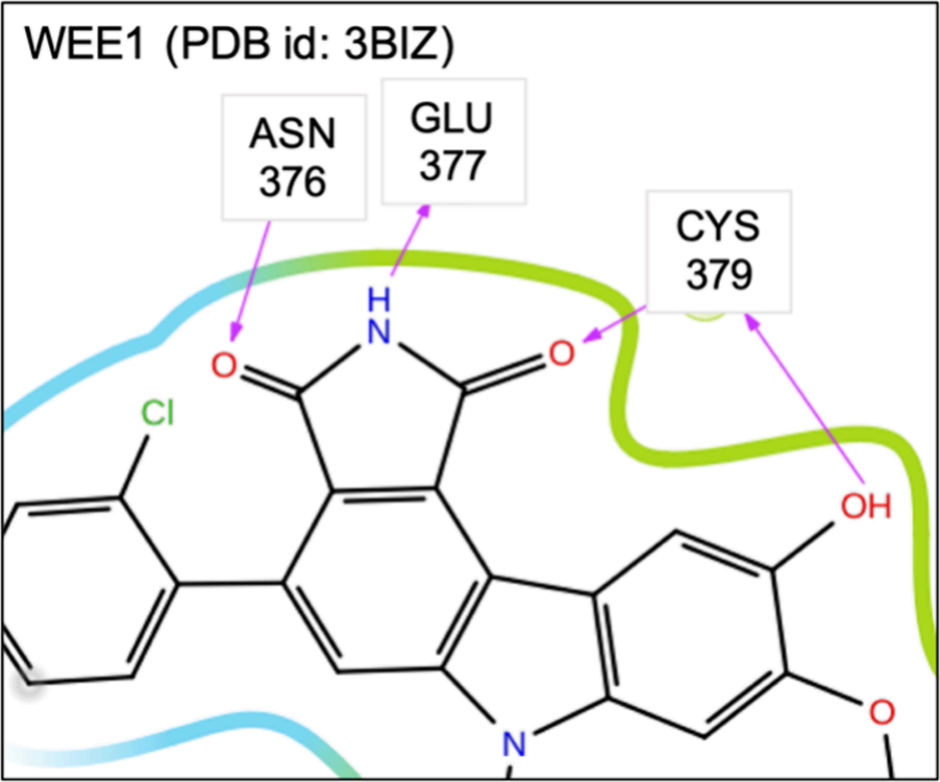
Two-dimensional (2D) diagrams of the interactions between ligand 61E and the binding site of WEE1 (3BIZ). Only the side chains and interactions relevant to the analysis are shown. This diagram was generated using Schrödinger Maestro. Full 2D diagram is provided in Figure S14b of the Supporting Information.

**Table 5:** Average Number of Water Molecule Neighbors (within 3.5 Å) of the Functional Groups of Side Chains That Formed Hydrogen Bonds with the Ligand in the Cognate Conformation of WEE1^a^

	ASN376-ND2	ASN376-OD1	GLU377-O	CYS379-SG	CYS379-O	CYS379-N	total H_2_O neighbors
*rigid*	1.92	0	1.00	0.57	1.09	1.19	5.77
*flexible*	0.19	1.55	1.01	0.67	1.04	1.04	5.50

a This is a metric of how well solvated each functional group is in the *rigid* and *flexible* simulations.

**Table 6:** Average Number of Hydrogen Bonds Formed between Water and the Functional Groups of the Side Chains That Formed Hydrogen Bonds with the Ligand in the Cognate Conformation of HMDH

	ASN376-ND2	ASN376-OD1	GLU377-O	CYS379-SG	CYS379-O	CYS379-N	total H-bonds
*rigid*	0.92	0	0.96	0.45	0.97	0.93	4.23
*flexible*	0.10	1.22	0.98	0.58	0.97	0.86	4.71

#### 3.5.3 System 3: HMDH (PDB ID: 3CCW)

In the prior sections, we outlined that there is a significant thermodynamic driving force for the protein to adopt conformations that are complementary to the water. Here, using HMDH as an example, we detail how, in the *flexible* simulations, the side chains proximal to the protein–ligand hydrogen bonds restructure when the ligand is not present. The left panel of Figure 15 shows the complex of ligand 4HI[Fn fn3] with HMDH. The binding site encloses the ligand carboxylate and hydroxy group, which form three hydrogen bonds with the protein (Figure 16). The middle and right panels show the solvent-accessible volume in the binding site (blue scaffold) for the *rigid* and *flexible* simulations and hydration sites that form hydrogen bonds with the same functional groups as the ligand.

**Figure 15:**
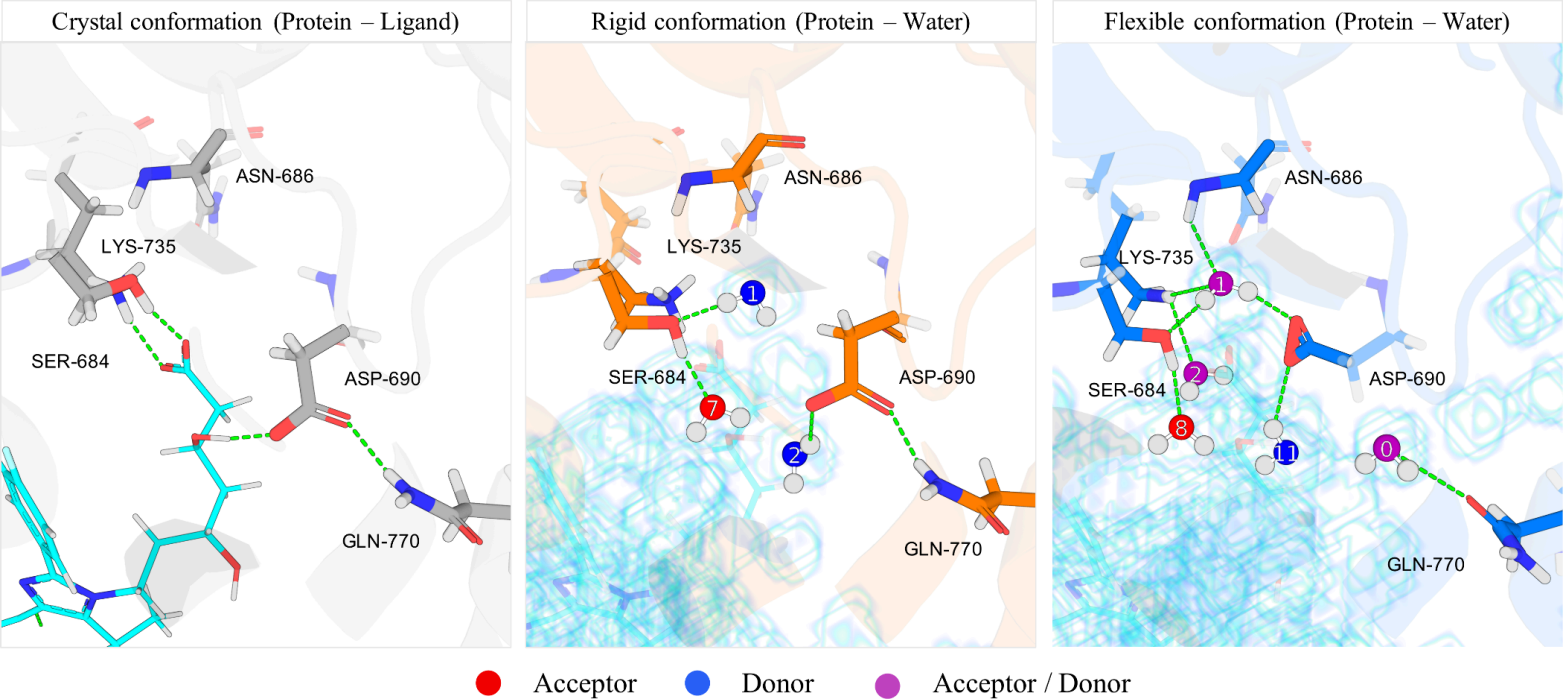
Binding cavity of HMDH. (Left) Ligand 4HI and the hydrogen bonds that it forms with the protein. (Middle) Solvent-accessible surface (scaffolded) and SSTMap hydration sites that interact comparably to the ligand in the *rigid* simulation. (Right) Same results for the *flexible* simulation with the time averaged structure of the protein. Hydration sites that donate hydrogen bonds to the surface are colored in blue. Those that accept are in red and those that both donate and accept are colored in purple.

**Figure 16:**
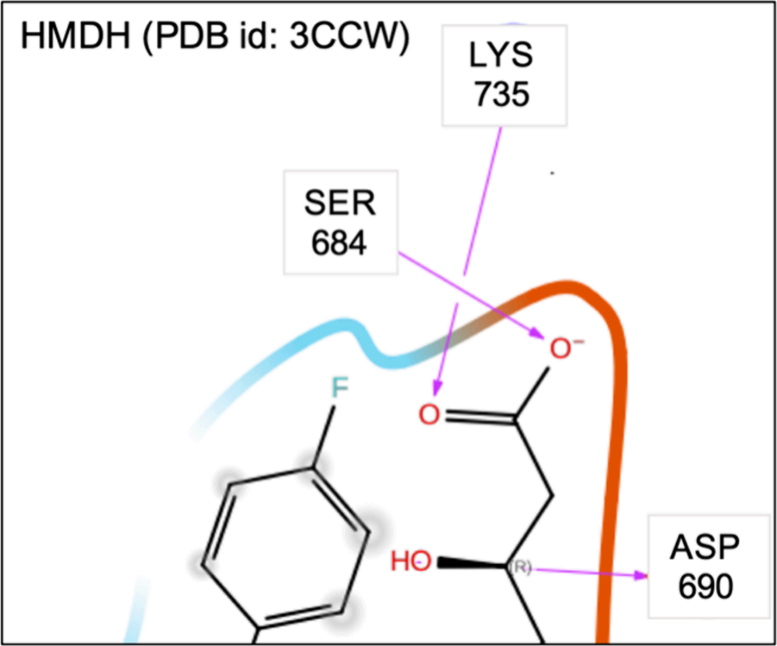
Two-dimensional (2D) diagrams of the interactions between ligand 4HI and the binding site of HMDH (3CCW). Only the side chains and interactions relevant to the analysis are shown. This diagram was generated using Schrödinger Maestro. Full 2D diagram is provided in Figure S14c of the Supporting Information.

The protein functional groups that are hydrogen-bonded with ligand 4HI are significantly better hydrated in the *flexible* simulations than in the *rigid*, forming approximately 2.5 more hydrogen bonds with water (Table 7) and having more than 2 additional water neighbors (Table 8).

**Table 7:** Average Number of Hydrogen Bonds Formed between Water and the Functional Groups of the Side Chains That Formed Hydrogen Bonds with the Ligand in the Cognate Conformation of HMDH (PDB ID: 3CCW)

	LYS735-NZ	SER684-OG	ASP690-OD1,2	total H-bonds
*rigid*	0.29	1.88	1.11	3.28
*flexible*	0.92	1.84	3.08	5.84

**Table 8:** Average Number of Water Molecule Neighbors (within 3.5 Å) of the Functional Groups of Side Chains That Formed Hydrogen Bonds with the Ligand in the Cognate Conformation of HMDH (PDB ID: 3CCW)­^a^

	LYS735-NZ	SER684-OG	ASP690-OD1,2	total H_2_O neighbors
*rigid*	1.04	2.88	1.75	5.67
*flexible*	1.90	2.72	4.13	8.75

a This is a metric of how well solvated each functional group is.

In order to make these hydrogen bonds, the carboxylate of ASP-690 rotates to interact with HS1 in the *flexible* simulations (Figure 15, right). This makes hydration site 1 (HS1), which is energetically unfavorable compared to the bulk in the *rigid* simulations, energetically *favorable* in the *flexible* simulations, lowering the energy by more than 2 kcal/mol. In the *rigid* system, HS1 is frustrated and only forms 1.34 hydrogen bonds with its surroundings, and in the *flexible* system, it forms a full complement (3.52) of hydrogen bonds with its surroundings (Table 9). Importantly, the carboxylate of ASP-690 forms a hydrogen bond with the ligand in the cognate structure; however, when the protein side chains are free to move in the MD simulations, the carboxylate moves to a position that is too distal to form a hydrogen bond with the ligand. We investigated this and found in our production trajectories what proportion of the frames were in the up position (Figure 15, right) and in the down position (Figure 15, middle). In all 20,000 simulation frames, the carboxylate was in the up position. In the *rigid* simulations and cognate structure, the amino group of GLN-770 forms a hydrogen bond with the carboxylate group of ASP-690. When this carboxylate repositions to interact with HS1 in the *flexible* simulations, it is no longer available to accept a hydrogen bond from GLN-770, and the rotamer state of GLN-770 flips in 99.95% of the simulation frames (10 out of 20,000 frames were in a position comparable to the cognate structure). In the *rigid* structure, the side chain of GLN-770 is not available to form hydrogen bonds in the binding cavity. Its amino group has no water neighbors and is not exposed to the binding pocket, and the carbonyl is flipped away from the pocket. In the *rigid* structure, however, the carbonyl flips to be exposed to the binding pocket and forms a hydrogen bond with HS0 (Tables 10 and S2).

**Table 9:** Protein–Water Interaction Energy (*E*_sw_), Water–Water Interaction Energy (*E*_ww_), Total Energy (*E*_tot_), and Average Number of Protein–Water Hydrogen Bonds of Hydration Site 1 in Each System^a^

	*E*_sw_	*E*_ww_	*E*_tot_	protein–water H-bonds
*rigid*	–5.45	–5.40	–10.85	1.34
*flexible*	–11.69	–1.23	–12.92	3.52

a The total energy values are referenced to the energy of the same number of neat water molecules as found in the hydration site.

**Table 10:** Average Number of Water Molecule Neighbors (within 3.5 Å) and Hydrogen Bonds Formed between Water and GLN770 of HMDH (PDB ID: 3CCW)

	H_2_O neighbors			H-bonds		
	GLN770-NE2	GLN770-OE1	total	GLN770-NE2	GLN770-OE1	total
*rigid*	0.00	1.00	1.00	0.00	0.98	0.98
*flexible*	1.00	2.22	3.22	0.91	1.80	2.72

These structural rearrangements are important when considering the complementarity of potential ligands to the protein surface. The *rigid* simulations were restrained from moving significantly from the cognate structure of the protein, which is complementary to the ligand. However, when the system is *flexible*, the position of the hydrogen bond acceptors of ASP-690 has moved, and a new hydrogen bonding acceptor from GLN-770 is revealed in the binding pocket. This makes the cognate ligand not complementary to the binding site generated in the *flexible* simulations. Importantly, the configurations of the protein that are complementary to the cognate ligand are never sampled in the 80 ns MD simulations; however, there may be ligands that are complementary to the structure revealed in the *flexible* simulations.

## 4 Discussion

The concept of protein–ligand complementarity is a fundamental premise of structure-based drug discovery and effectively states that tightly bound ligands make complementary contacts with the protein by donating or accepting hydrogen bonds and making hydrophobic contacts where appropriate. Correspondingly, the concept of induced fit^59^ suggests that protein binding sites conform their shapes to the ligands in response to the ligand’s presence. Here, we discuss the concept of *protein–water complementarity*. Our results suggest that when a protein is in its ligand-bound conformations, it cannot form optimal hydrogen bond interactions with water, and correspondingly, the solvation energetics provide a considerable driving force to restructure the binding cavity. In every system investigated in this study, when the *rigid* restraints on the side chains were absent, the protein binding sites adopted alternate conformations, which formed a greater number of hydrogen bonds with water and had more favorable protein–water interactions. Thirty-three of the 34 systems have a lower overall energy of solvation, effectively adopting binding site conformations that are *complementary* to the water molecules in the binding site.

These results indicate that the protein incurs a significant solvation energetic cost in forming binding site conformations that are *complementary* to small-molecule chemical compounds. The average cost is substantial and was estimated here to be approximately 14.43 kcal/mol, with the estimations ranging from system to system, from −3.82 kcal/mol for PPAR-δ to 47.00 kcal/mol for ACE (Figure 5). Consistent with the idea of induced fit, this cost may prevent the apoprotein from sampling conformations that are complementary to tightly binding ligands. Indeed, in simulations of HMDH (PDB id: 3CCW) and PPAR-γ (PDB id: 2GTK), the unligated simulations rarely or never sampled the cognate conformations that were complementary to the bound ligands.

We believe the results have significant implications in both the search for cryptic pockets and with regard to the proper use of solvation mapping methods in molecular recognition. We briefly discuss each of these in the following subsections.

### 4.1 Implications for Cryptic Pocket Identification

Due to the inherent flexibility of proteins, binding cavities change shape affecting the volume, surface accessibility, and the positions of hydrogen bond and hydrophobic interaction sites. This is exciting for drug discovery programs, as differently shaped cavities will correspondingly bind different chemical compounds, providing design inroads to potential improvements of ADMET properties, selectivity, and patentability over known compounds. For these reasons, significant efforts, both computational and experimental, have been dedicated to identifying alternate druggable conformations of binding pockets other than those already known from experimental structures.

Experimentally, studies using methods such as NMR,^60–62^ Cryo-EM^63–66^ and room-temperature crystallography^67–69^ have focused on revealing alternate conformations of proteins and identifying such “cryptic” pockets. Computational methods have focused on simulation sampling-based approaches that improve the exploration of protein conformational free-energy surfaces using enhanced sampling methods, such as metadynamics,^70–73^ umbrella sampling,^74–76^ and replica exchange molecular dynamics (REMD),^77,78^ or machine learning approaches, such as CryptoSite^79^ and incarnations of AlphaFold.^80–83^

The results described here describe a significant solvation energetic cost for unligated proteins to adopt conformations that are complementary to potential or known ligands. The magnitude of this cost suggests that in many cases, there may be little to no overlap between the binding site configurational ensembles of unligated proteins with those of ligated proteins. In two of the three systems that bind multidentate ligands (HMDH and PPAR-γ), we found that the conformations that are complementary to the cognate ligands were rarely, if ever, sampled in unbiased molecular dynamics simulations. Other computational approaches aimed at sampling the conformational space of unligated binding pockets may also rarely, if ever, sample conformations that can tightly bind chemical compounds. Experimental methods suffer from the same problems. The results here suggest that because of solvation-free energetic costs, unligated proteins will rarely be in conformations that are complementary to potential ligands and will therefore have a minimal, likely unresolvable, signal in NMR, X-ray, or Cryo-EM experiments.

The results also suggest that computational techniques that explore conformations with chemical compounds present (such as mixed solvent MD or high-throughput fragment-based experimental methods) may have a better chance of finding relevant binding pocket conformations than methods that explore the free energy landscape of unligated proteins.^84^ This is also likely true for biased computational methods developed to improve sampling in molecular dynamics simulations. These methods are generally designed to overcome free energy barriers and better sample regions about the minima on free energy surfaces. What is described here, however, are energetic costs to generating these conformations and therefore likely not in the region of the free energetic minima.

We note that the lack of conformational overlap may apply only to a subset of proteins. In WEE1, which is characterized as a relatively unenclosed binding site, there seemed to be significant overlap in the apo- and ligand-bound conformations, and conformations that bound the cognate ligand were explored.

### 4.2 Implications for the Use of Solvation Mapping Methods in Drug Discovery

The results presented here have an impact on how solvation thermodynamic mapping methods are best used. Most methods aimed at computationally estimating the thermodynamics of solvating binding sites rely upon an analysis of the solvation of *rigid* cognate ligand-bound protein structures for which the ligand has been removed.^4–7,12,13,15–21,48,85,86^

In particular, solvation mapping methods using IST have been widely used to estimate the solvent contribution to the binding of ligands or the differences in the binding affinity of congeneric pairs of ligands. Many of these applications have relied upon the supposition that the main contribution to binding is due to the displacement of water from the binding site and that the solvent contribution can be estimated by the difference in thermodynamic properties of displaced water in the binding site and in neat water to which the water has presumably been displaced.^4,48,49,87–91^ The maps used in this solvent displacement approximation are generally generated from simulations of *rigid* cavities. As the solvation of *rigid* binding cavities is significantly less favorable than the solvation of *flexible* cavities, the results here suggest that this yields a significant overestimation of the contribution of binding. Along with the energetics, the structure of water often differs considerably between *flexible* and *rigid* cavities. Approaches that estimate the contribution of displacing water from hydration sites may also be flawed as the positions and thermodynamics of the hydration sites often change between *rigid* and *flexible* cavities (e.g., see Figure 15, HMDH).

In summary, this work demonstrates that the energetic cost of solvation poses a substantial barrier for unligated binding sites to adopt configurations that are complementary to those of their cognate ligands. Across a diverse set of systems, we find that flexible binding sites are significantly better solvated than their rigid, ligand-complementary counterparts, on average by 14.43 kcal/mol, highlighting a strong energetic preference for protein–water over protein–ligand complementarity in the absence of the ligand. These solvation-driven energetic penalties not only limit the configurational overlap between ligated and unligated proteins but also imply that apo structures may systematically fail to reveal cryptic or ligand bindable conformations. Moreover, our findings expose fundamental limitations in solvation mapping methodologies that assume the rigidity of the binding site, which can lead to considerable overestimation of water displacement contributions to binding affinity. Together, these insights underscore the need for approaches that explicitly account for environment-dependent differences in protein conformational sampling, whether in the presence of the solvent or ligand, when assessing ligandability and guiding structure-based drug discovery.

## Supplementary Material



## Data Availability

GIST, SSTMap data, and MD input files are available on the Kurtzmanlab Github located at: https://github.com/KurtzmanLab/BindingSiteSolvation

## Footnotes

a (2*s*)-3-(1-{[2-(2-Chlorophenyl)-5-methyl-1,3-oxazol-4-yl]­methyl}-1h-indol-5-yl)-2-ethoxypropanoic acid.

b 4-(2-chlorophenyl)-8-[3-(dimethylamino)­propoxy]-9-hydroxy-6-methylpyrrolo­[3,4-*c*]­carbazole-1,3­(2*H*,6*H*)-dione.

c (3*R*,5*R*)-7-[4-(Benzylcarbamoyl)-2-(4-fluorophenyl)-5-(1-methylethyl)-1*H*-imidazol-1-yl]-3,5-dihydroxyheptanoic acid.

## Notes

List of systems investigated and PDB IDs; solvation energetic and hydrogen bond data for each individual system studied; examples of bidentate ligands; and full protein–ligand contact maps for PPAR-γ, WEE1, and HMDH (PDF)
