# Supplementary material for: Solvation Energetic Costs of Cognate Binding Site Formation
Source: J Chem Inf Model. 2025 Aug 15;65(17):9177–95. doi: 10.1021/acs.jcim.5c01432 (PMC12421683; doi:10.1021/acs.jcim.5c01432)
Supplement: Supplementary file 1 [file ci5c01432_si_001.pdf]

Solvation Energetic Costs of Cognate Binding Site Formation

Yeonji Ji[1], *Vjay Molino*[1], *Steven Ramsey*[2], *Tom Kurtzman*[1,2,3]

[1] Ph.D. program in Biochemistry, The Graduate Center, City University of New York, New York, USA

[2] Department of Chemistry, Lehman College, City University of New York, Bronx, USA

[3] Ph.D. Programs in Biology & Chemistry, The Graduate Center, City University of New York, New York, USA

Table S1. 34 DUD-E systems implicated in the study.

Target Name	PDB ID	Chain	Description	Ligand ID
ACE	3BKL	A	Angiotensin-converting enzyme	KAW
ADRB1	2VT4	A	Beta-1 adrenergic receptor	P32
AKT1	3CQW	A	Serine/threonine-protein kinase AKT	CQW
BRAF	3D4Q	A	Serine/threonine-protein kinase B-raf	SM5
DEF	1LRU	A	Peptide deformylase	BB2
FGFR1	3C4F	A	Fibroblast growth factor receptor 1	C4F
FPPS	1ZW5	A	Farnesyl diphosphate synthase	ZOL
GCR	3BQD	A	Glucocorticoid receptor	DAY
GRIK1	1VSO	A	Glutamate receptor ionotropic kainate 1	AT1
HDAC2	3MAX	A	Histone deacetylase 2	LLX
HMDH	3CCW	A, B	HMG-CoA reductase	4HI
HXK4	3F9M	A	Hexokinase type IV	MRK
KITH	2B8T	A	Thymidine kinase	THM
MAPK2	3M2W	A	MAP kinase-activated protein kinase 2	L8I
MCR	2AA2	A	Mineralocorticoid receptor	AS4
MK10	2ZDT	A	c-Jun N-terminal kinase 3	19A
NOS1	1QW6	A	Nitric-oxide synthase, brain	3AR
NRAM	1B9V	A	Neuraminidase	RA2
PARP1	3L3M	A	Poly [ADP-ribose] polymerase-1	A92
PGH2	3LN1	A	Cyclooxygenase-2	CEL
PLK1	2OWB	A	Serine/threonine-protein kinase PLK1	626
PPARA	2P54	A	Peroxisome proliferator-activated receptor alpha	735
PPARD	2ZNP	A	Peroxisome proliferator-activated receptor delta	K55
PPARG	2GTK	A	Peroxisome proliferator-activated receptor gamma	208
PTN1	2AZR	A	Protein-tyrosine phosphatase 1B	982
PUR2	1NJS	A	GAR transformylase	KEU
ROCK1	2ETR	A	Rho-associated protein kinase 1	Y27
SAHH	1LI4	A	Adenosylhomocysteinase	NOC
SRC	3EL8	A	Tyrosine-protein kinase SRC	PD5
THRB	1YPE	H	Thrombin	UIP
TRY1	2AYW	A	Trypsin I	ONO
UROK	1SQT	A	Urokinase-type plasminogen activator	UIW
WEE1	3BIZ	A	Serine/threonine-protein kinase WEE1	61E
XIAP	3HL5	A	Inhibitor of apoptosis protein 3	9JZ

Table S2. The average number of water molecule neighbors (within 3.5 Å) of the functional groups of side chains that formed hydrogen bonds with the ligand in the cognate conformation of HMDH. This is a metric of how well solvated each functional group is.

	LYS735-NZ	SER684-OG	ASP690-OD1,2	**Total H_2O Neighbors**
Rigid	1.04	2.88	1.75	5.67
Flexible	1.90	2.72	4.13	8.75

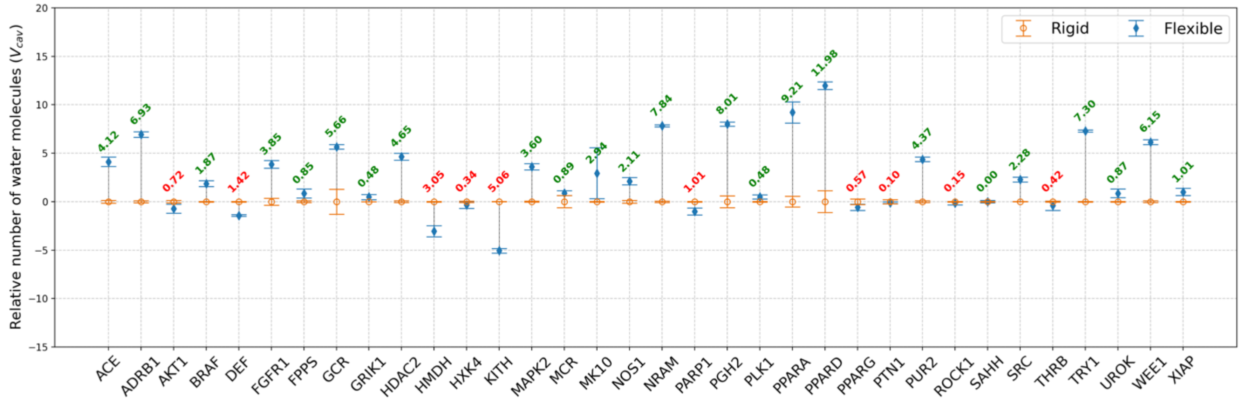

Figure S1. Relative number of water molecules in V_{cav}, for the flexible binding pockets to the rigid binding pockets for all 34 systems. The text shows the difference between *rigid* and *flexible* with green text denoting that the flexible cavities have more water molecules and red text denoting that the flexible cavities have fewer.

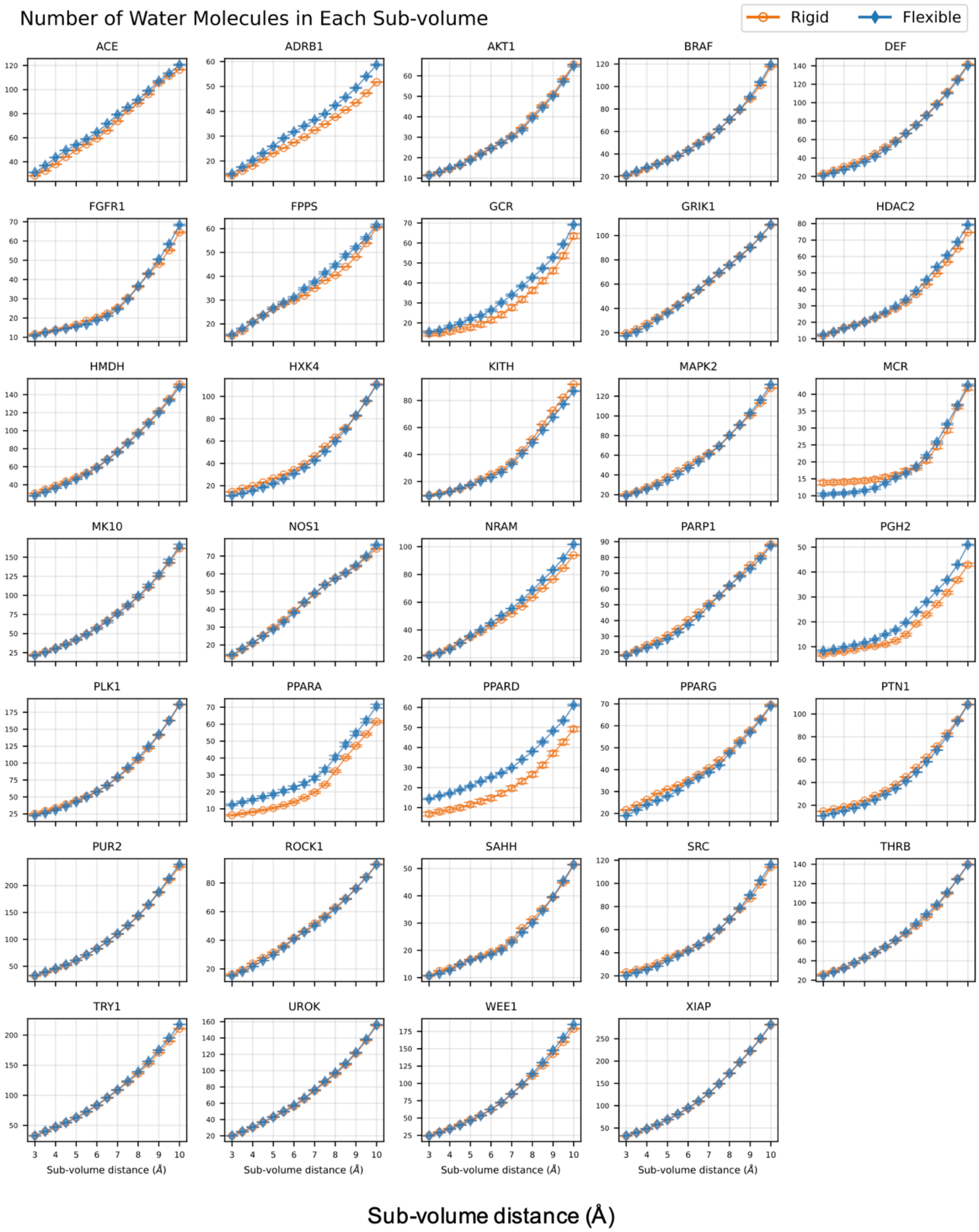

Figure S2. Number of water molecules in the *rigid* and *flexible* binding pockets (3-10 Å sub-volume) for 34 systems.

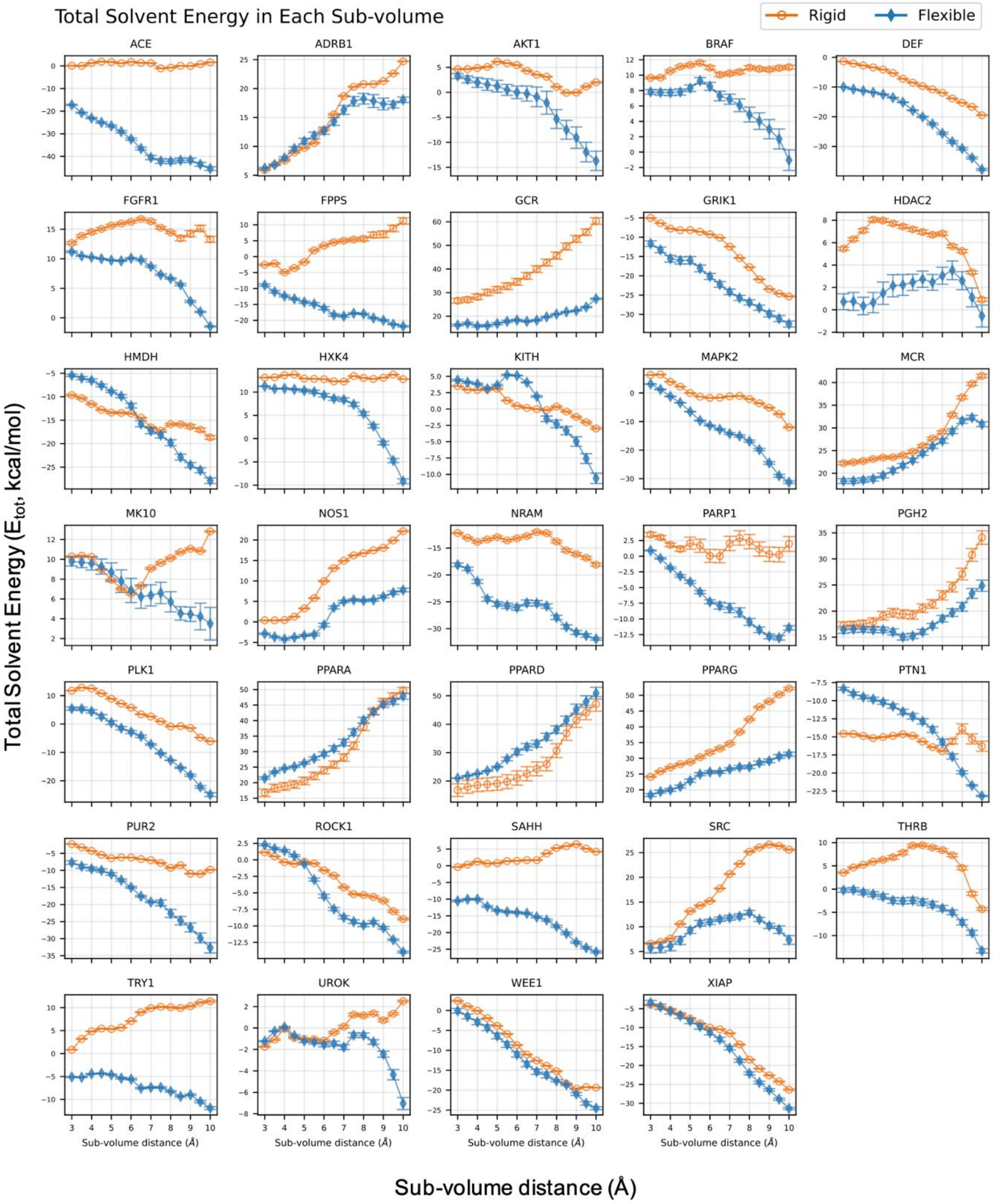

Figure S3. The total solvent energy (E_{tot}) for the *rigid* and *flexible* binding pockets (3-10 Å sub-volume) for 34 systems.

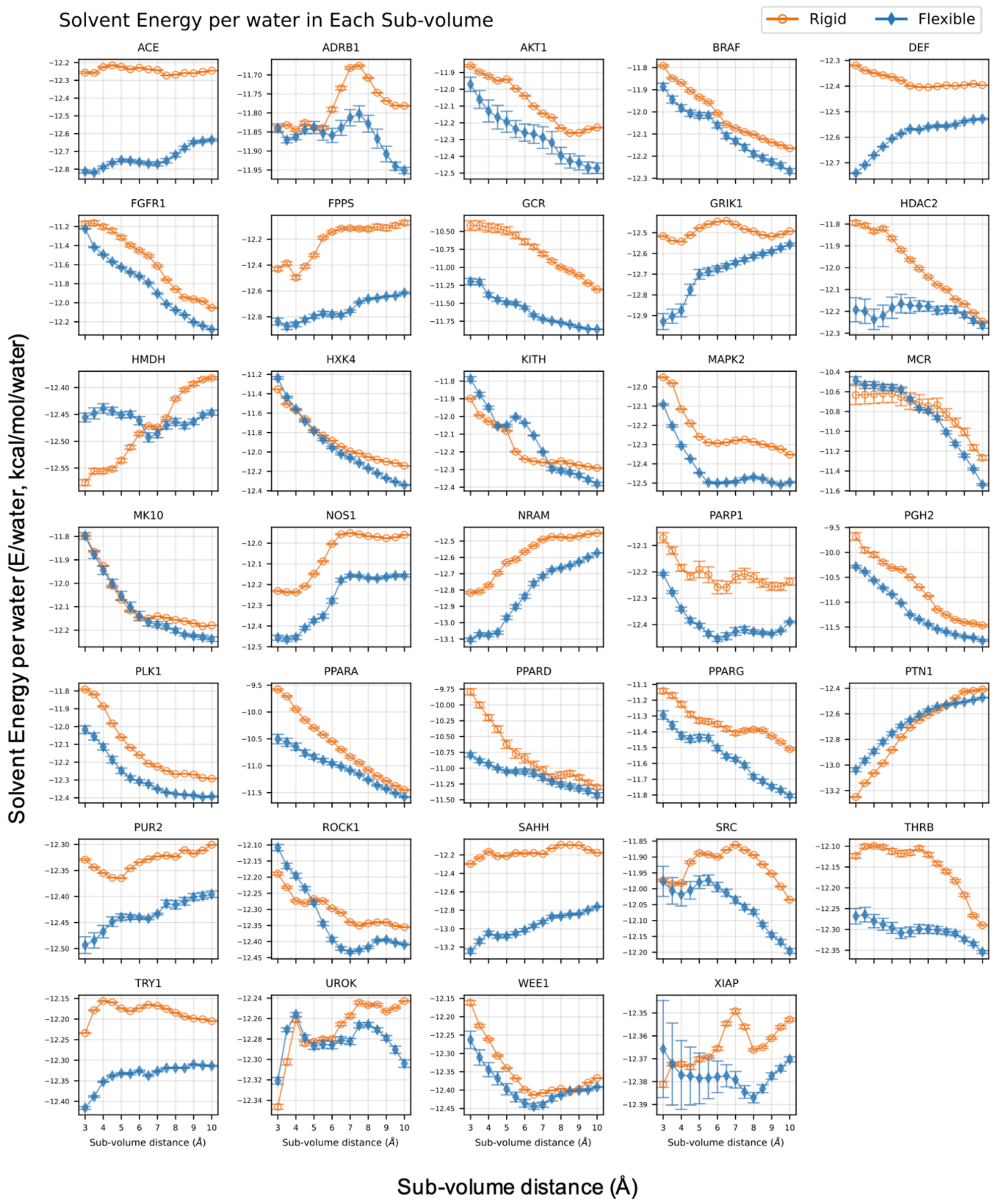

Figure S4. The solvent energy per water molecule (E/water) for the *rigid* and *flexible* binding pockets (3-10 Å sub-volume) for 34 systems.

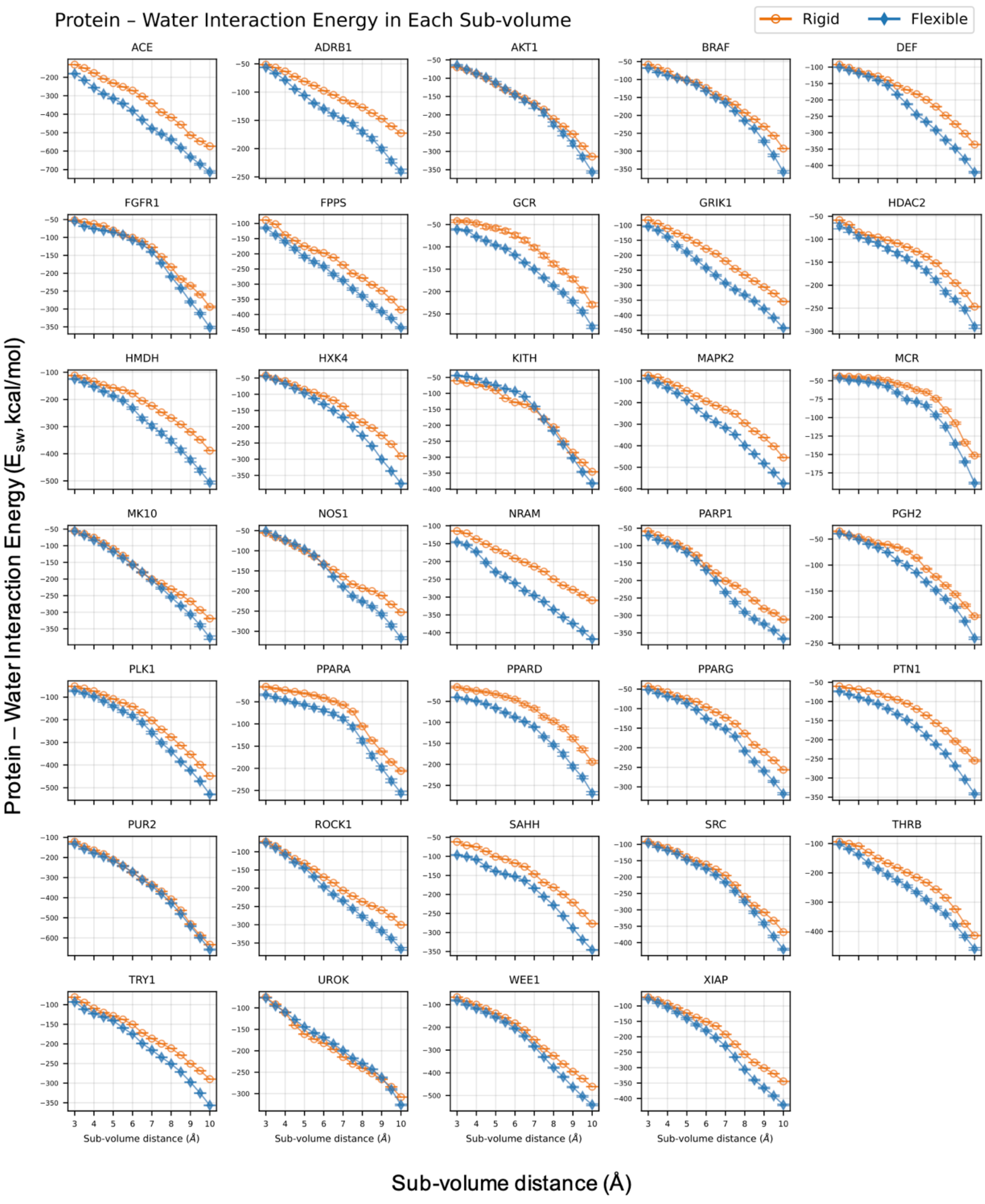

Figure S5. The protein-water energy (E_{sw}) for the *rigid* and *flexible* binding pockets (3-10 Å sub-volume) for 34 systems.

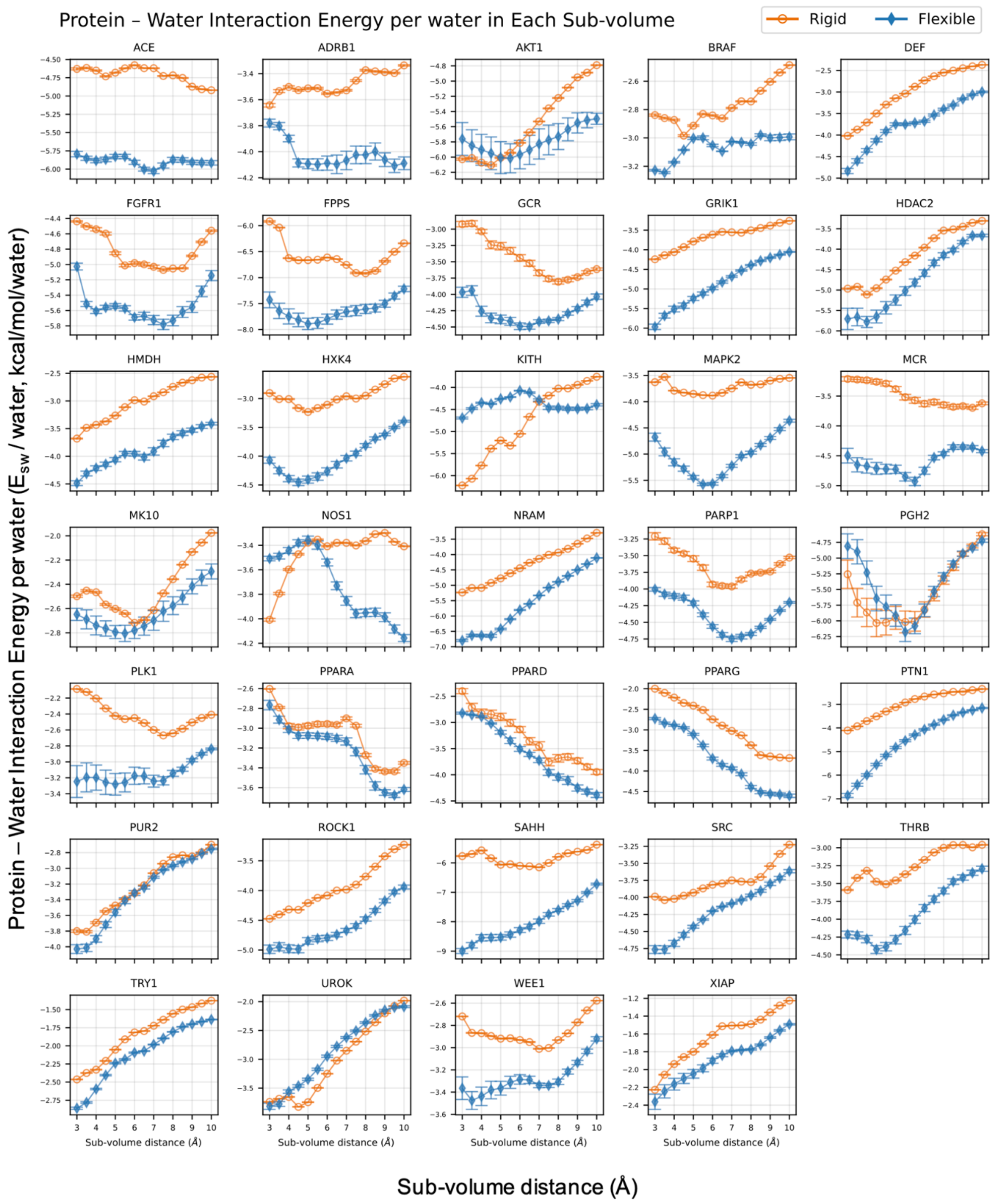

Figure S6. The protein-water energy per water molecule (E_{sw}/water) for the *rigid* and *flexible* binding pockets (3-10 Å sub-volume) for 34 systems.

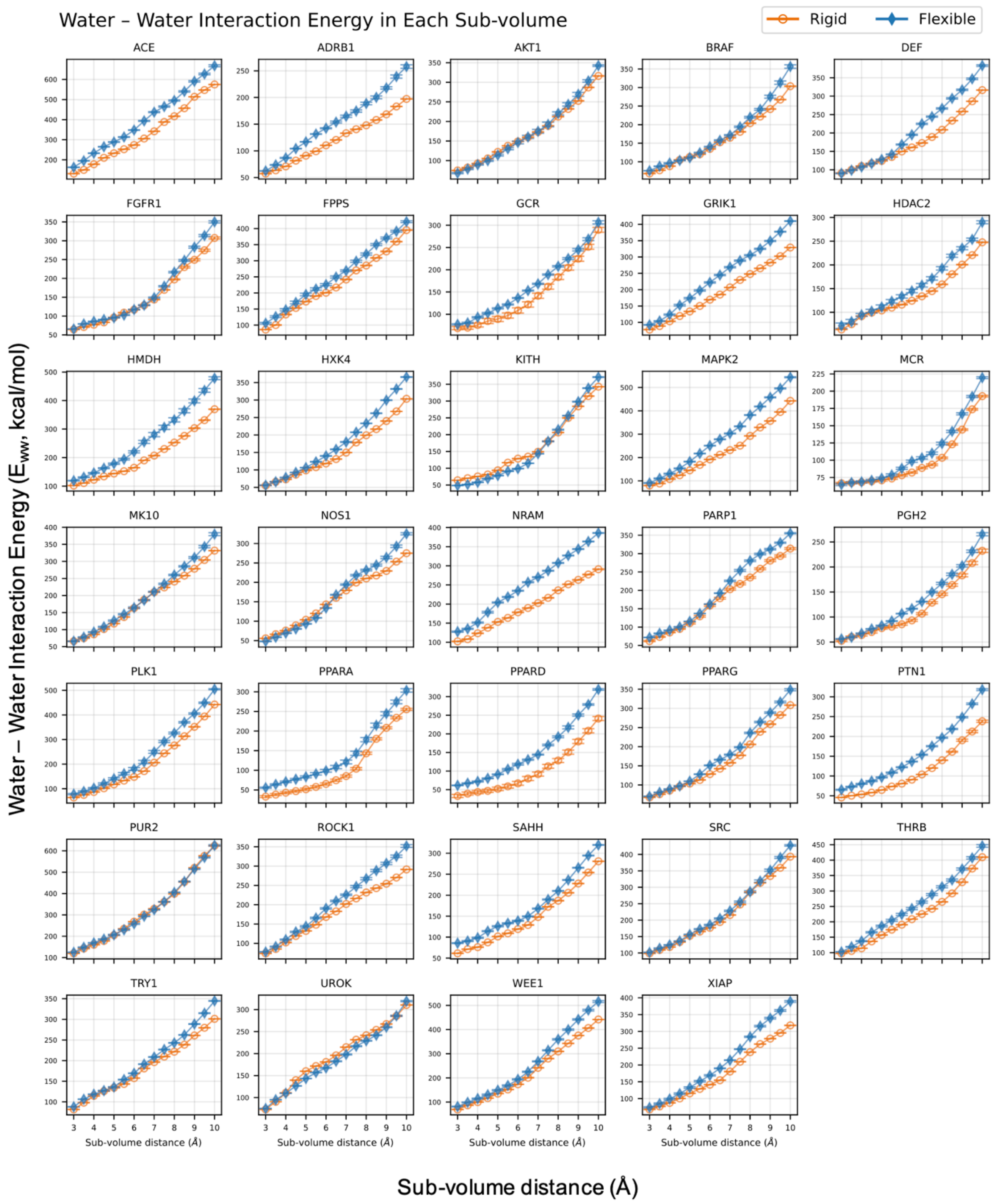

Figure S7. The water-water energy (E_{ww}) for the *rigid* and *flexible* binding pockets (3-10 Å sub-volume) for 34 systems.

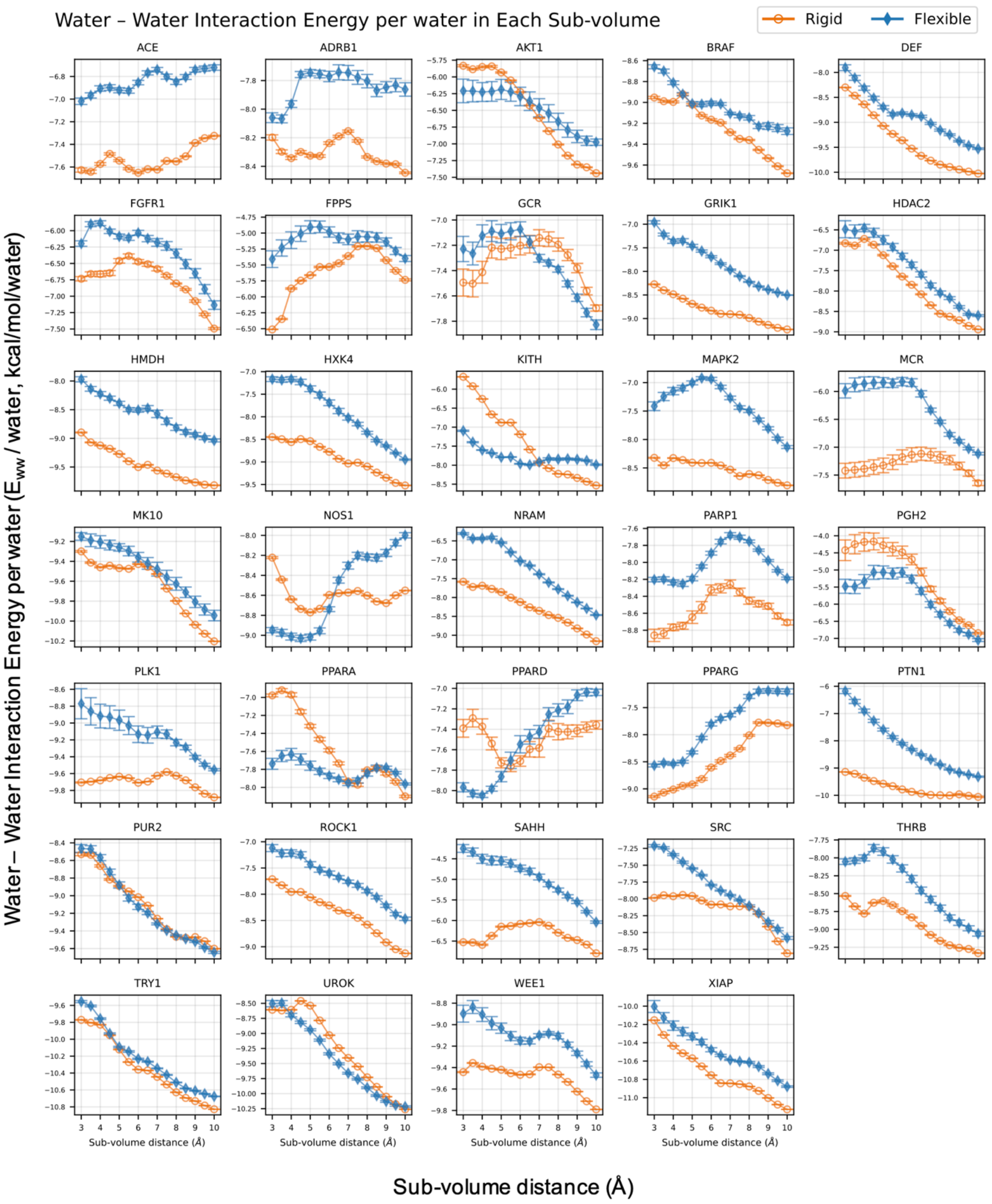

Figure S8. The water-water energy per water molecule (E_{ww}/water) for the *rigid* and *flexible* binding pockets (3-10 Å sub-volume) for 34 systems.

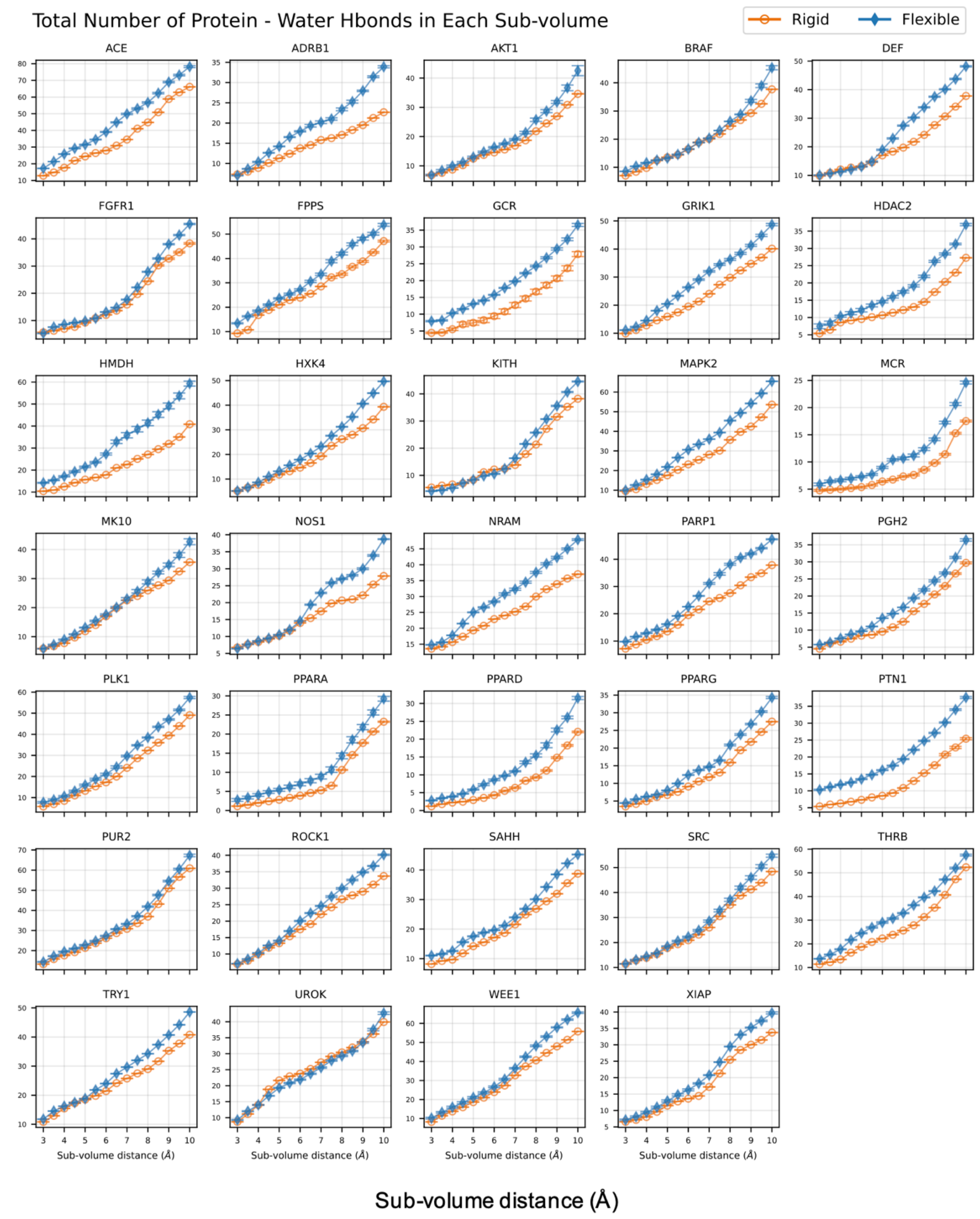

Figure S9. Number of protein-water hydrogen bonds for the *rigid* and *flexible* binding pockets (3-10 Å sub-volume) for 34 systems.

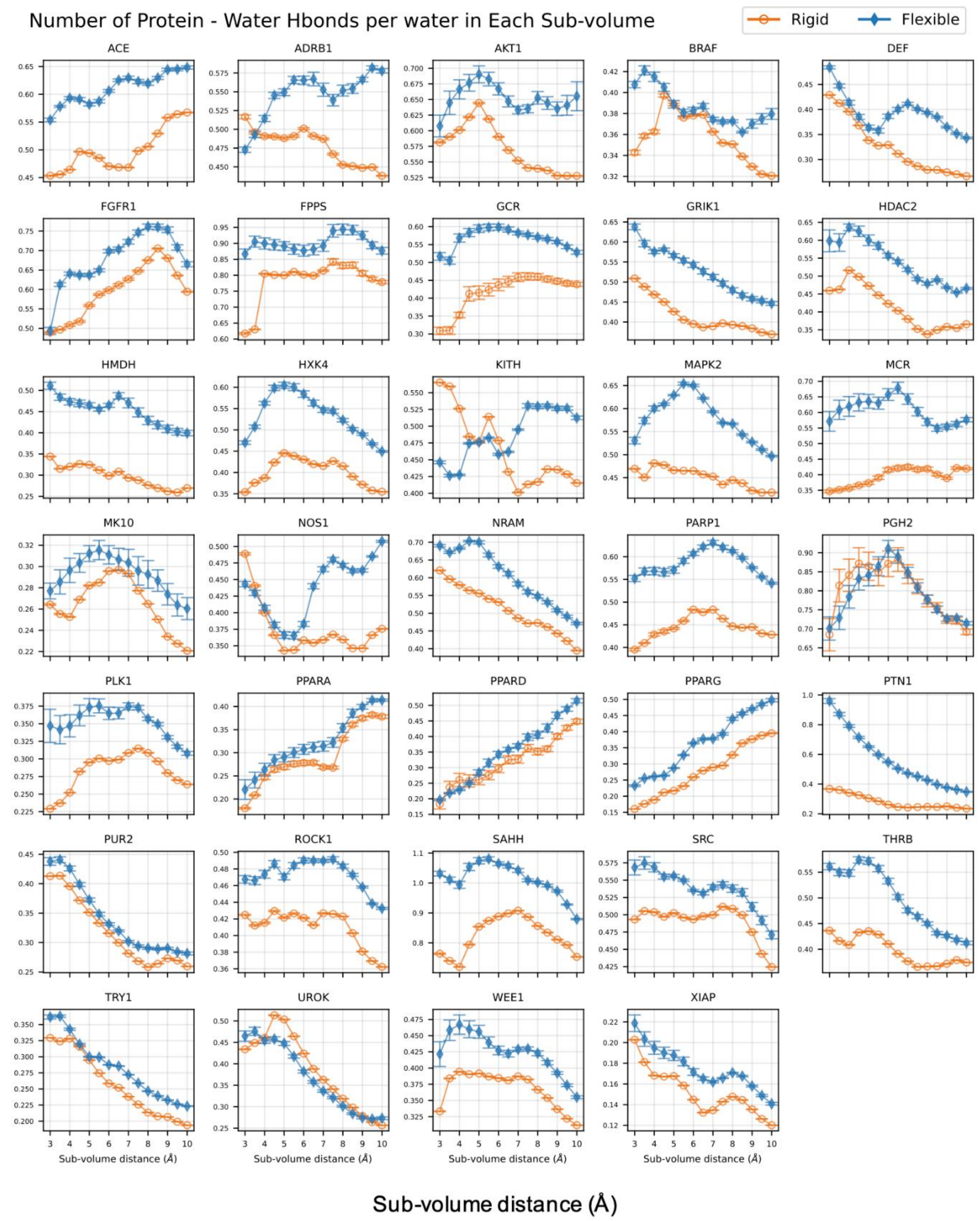

Figure S10. Number of protein-water hydrogen bonds per water molecule for the *rigid* and *flexible* binding pockets (3-10 Å sub-volume) for 34 systems.

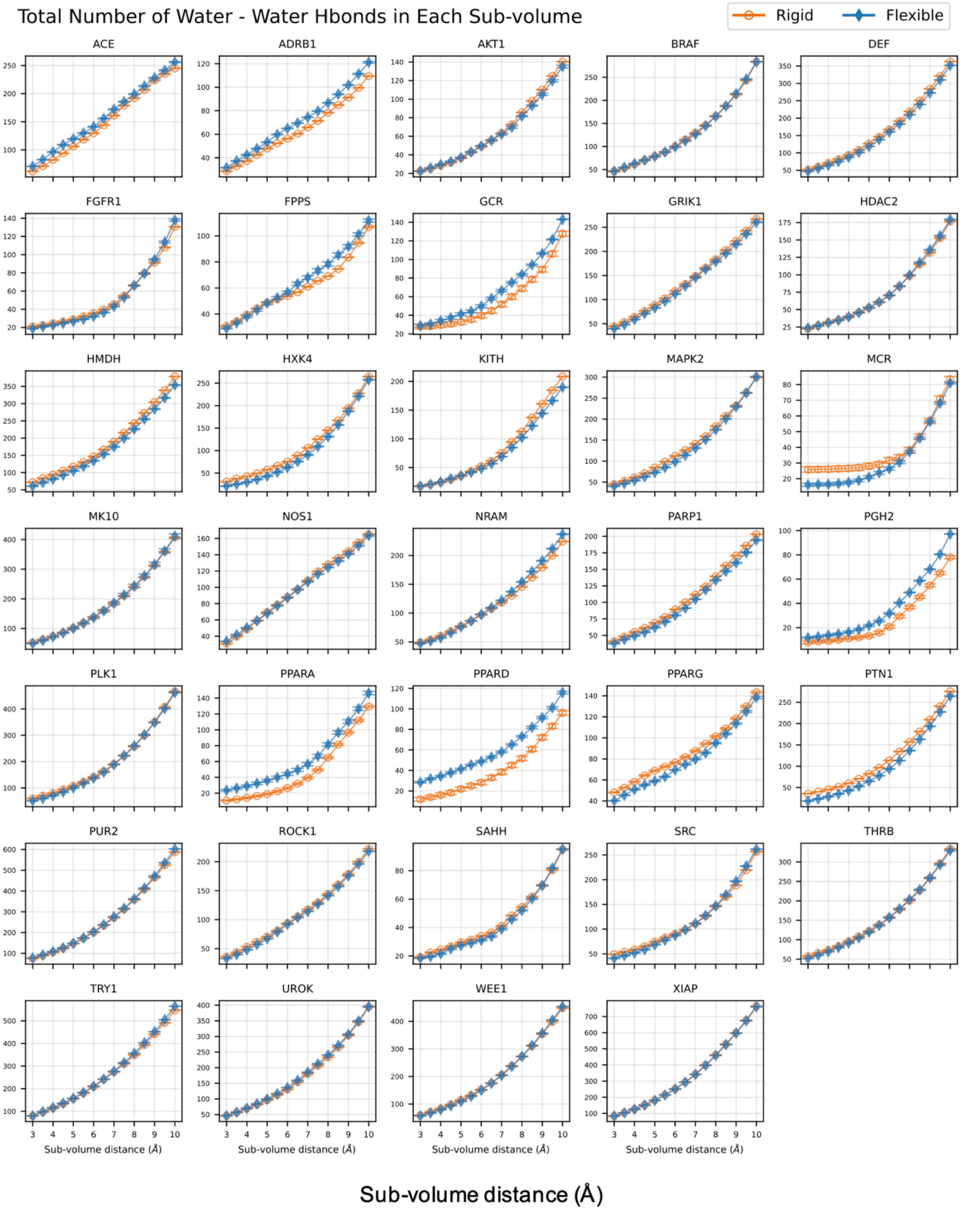

Figure S11. Number of water-water hydrogen bonds for the *rigid* and *flexible* binding pockets (3-10 Å sub-volume) for 34 systems.

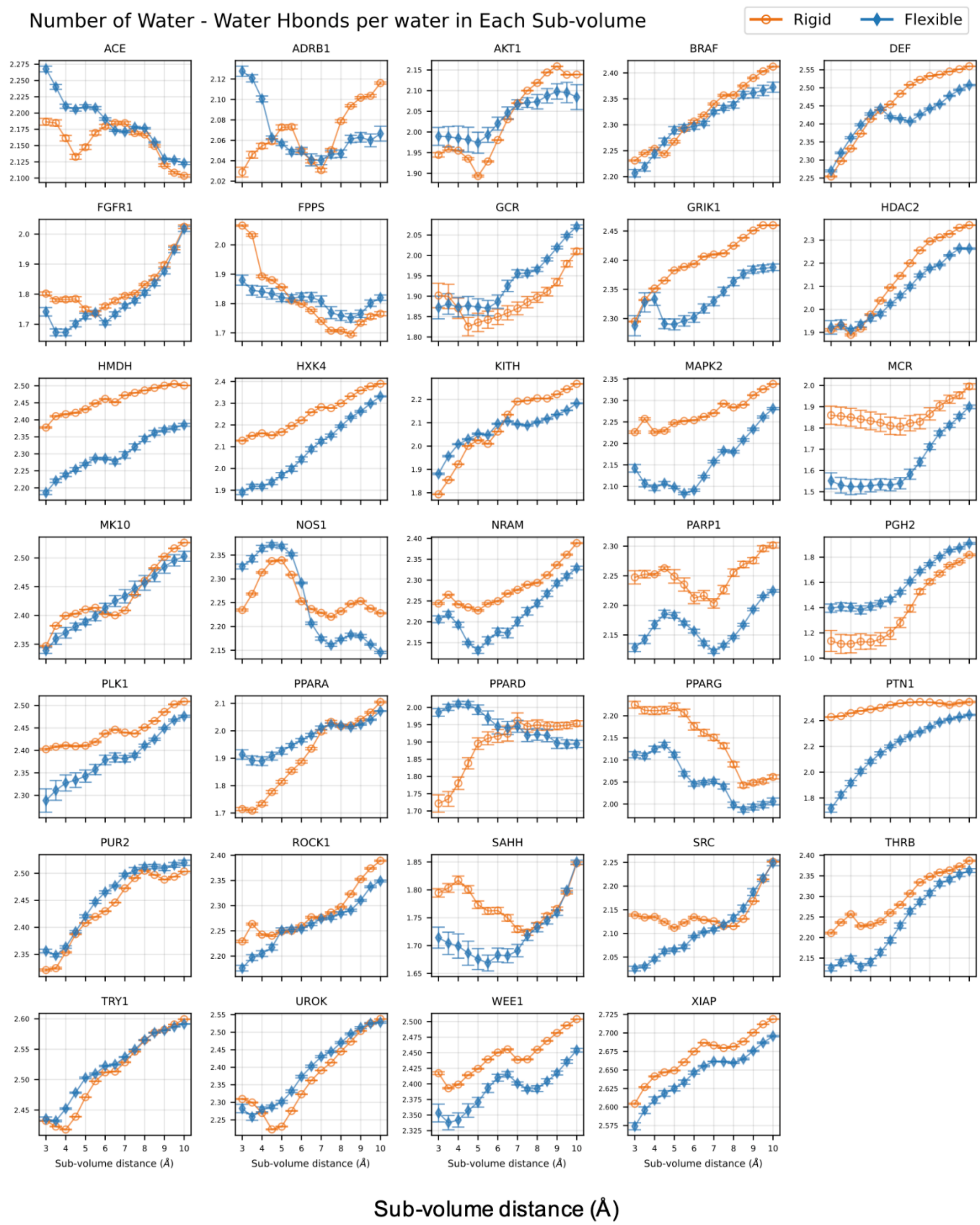

Figure S12. Number of water-water hydrogen bonds per water molecule for the *rigid* and *flexible* binding pockets (3-10 Å sub-volume) for 34 systems.

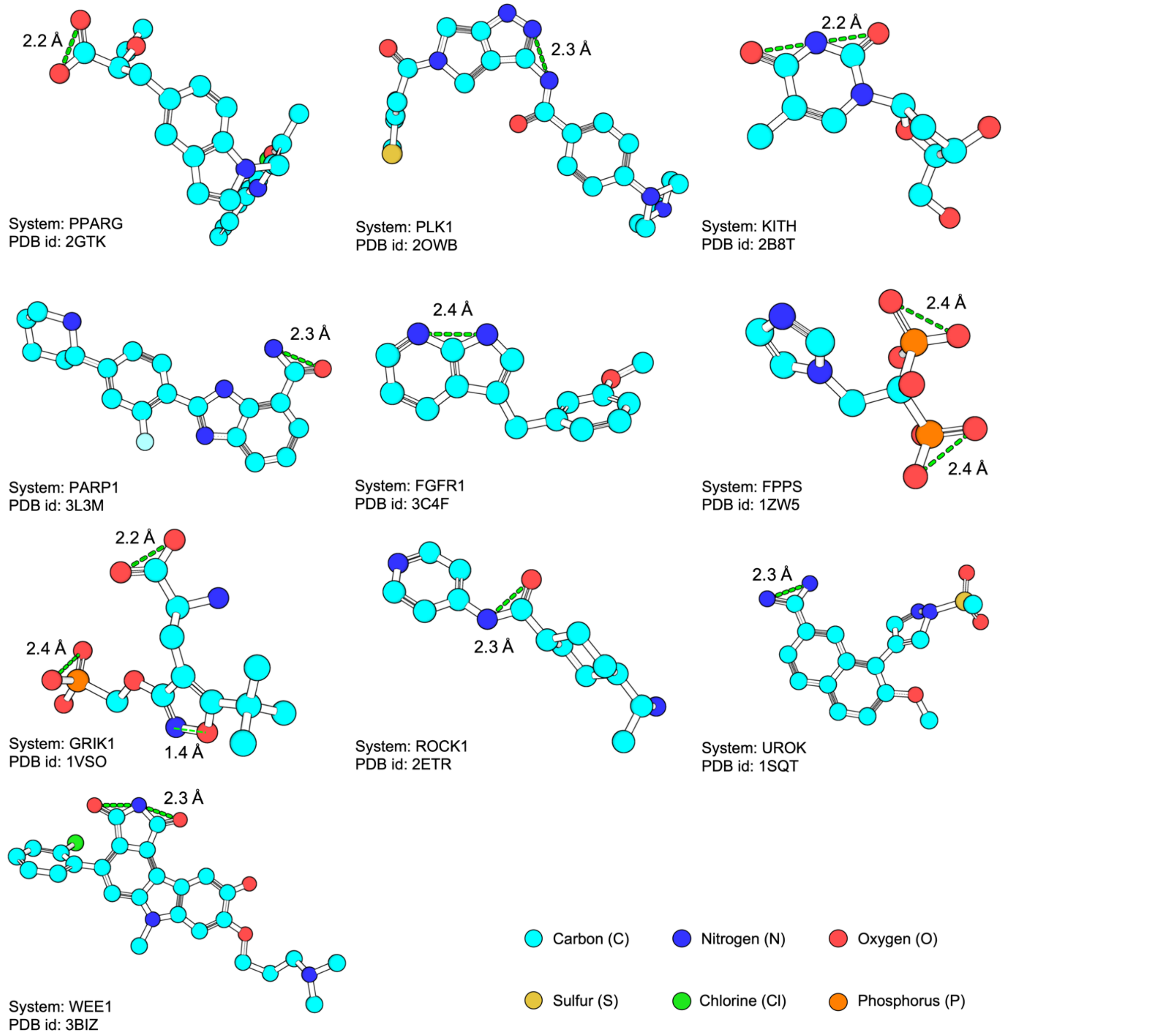

Figure S13. Example of bidentate ligands from the 34 systems investigated that have donor-acceptor pairs that are within 2.4 Å or less. Green dashed lines show the donor acceptor pairs and the distances in Å are correspondingly labeled.

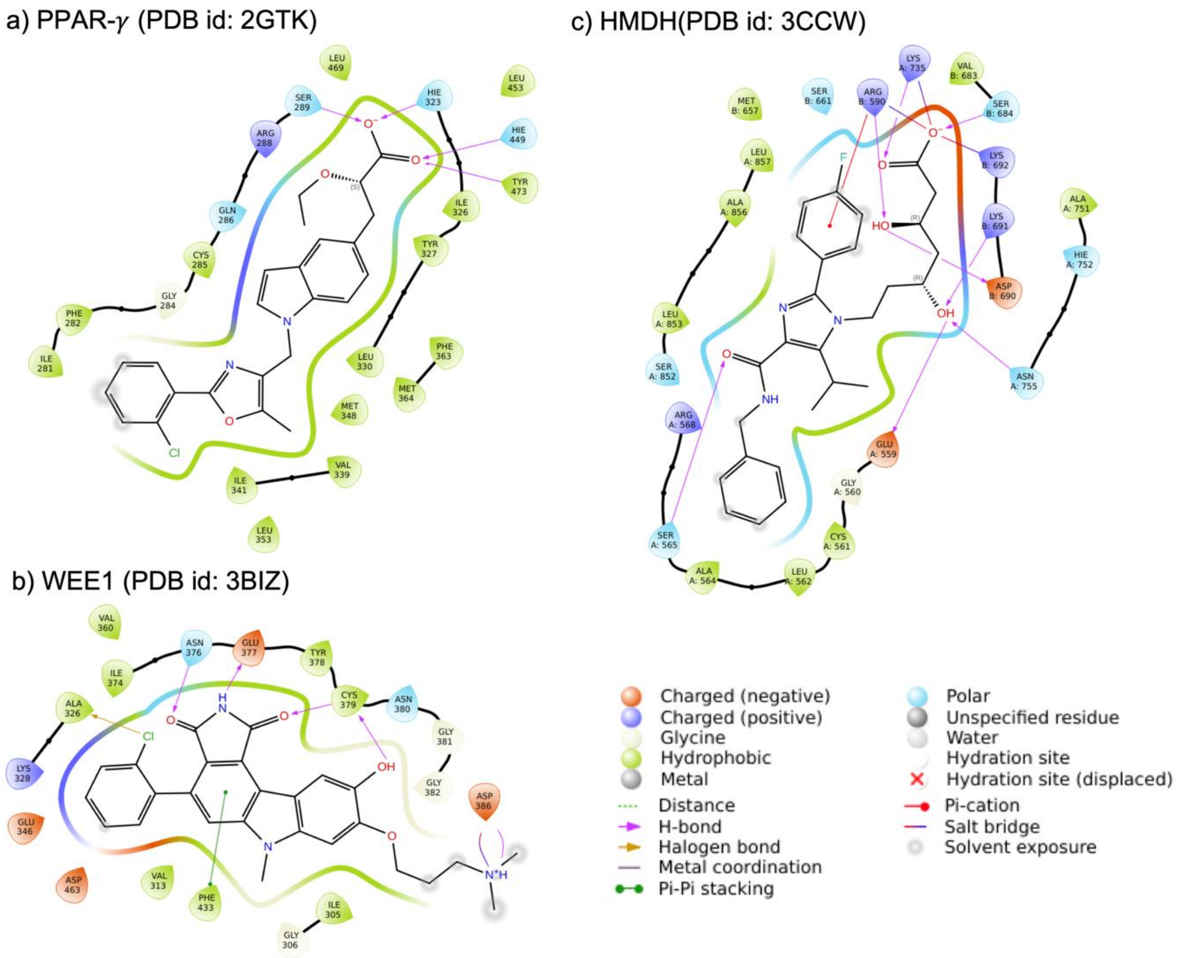

Figure S14. Full two-dimensional (2D) diagrams of protein-ligand interactions within the binding site of three systems a) PPAR-γ, b) WEE1, and c) HMDH.
